# Durability assessment of MgO/hydromagnesite mortars—Resistance to chlorides and corrosion

**DOI:** 10.1617/s11527-025-02765-z

**Published:** 2025-09-20

**Authors:** Fabio Enrico Furcas, Alexander German, Frank Winnefeld, Pietro Lura, Ueli Angst

**Affiliations:** 1 ETH Zürich, Institute for Building Materials, 8093 Zurich, Switzerland; 2 Empa, Swiss Federal Laboratories for Materials Science and Technology, Laboratory for Concrete and Asphalt, 8600 Dübendorf, Switzerland

**Keywords:** Mg-binders, Low-carbon, Durability, Chloride ingress, Corrosion, Moisture transport

## Abstract

The durability of MgO/hydromagnesite mortars was studied with respect to their corrosion performance and resistance to chloride attack and moisture. Rapid chloride ingress measurements showed chloride migration coefficients of ${D}_{\text{Cl}}={1\times 10}^{-13}$ to ${1\times 10}^{-12}$ ${\text{m}}^{2}/{\text{s}}$. MgO/hydromagnesite pastes were cured in alkaline chloride solution to investigate the formation of Mg-chlorides; however, no such phases were observed. The corrosion rate of carbon steel embedded in MgO/HY mortars, as determined by linear polarization resistance measurements, was in the range ${i}_{\text{corr}}=1\times {10}^{-9}$ $\text{A/}{\text{cm}}^{2}$ in dry and $1\times {10}^{-7}$ $\text{A/}{\text{cm}}^{2}$ in wet conditions, irrespective of the mortar composition or curing condition. These findings corroborate the hypothesis that, in the absence of chlorides, the moisture condition is the primary predictor of corrosion rate of carbon steel in the MgO/hydromagnesite binder. These accelerated, short-term experiments suggest that the binder may be suited to protect embedded carbon steel from corrosion under specific exposure conditions of practical relevance.

**Supplementary Information:**

The online version contains supplementary material available at 10.1617/s11527-025-02765-z.

## Introduction

MgO/hydromagnesite (MgO/HY) binders have been recently investigated as an alternative to traditional Portland cement clinker, prospectively associated with a lower environmental footprint [1–4]. Their main component, reactive MgO, can be produced from Mg-silicates [5–7] or Mg-rich brines [8, 9], thereby eliminating the intrinsic CO_2_ emissions associated with the calcination of MgCO_3_. Hydration of MgO in presence of hydromagnesite (Mg_5_(CO_3_)_4_(OH)_2_·4H_2_O – abbreviated as HY) leads to formation of a *hydrous carbonate*-containing *brucite* (abbreviated as HCB), which, being the sole hydration product in the MgO/HY binder, leads to strengthening in the hydrated binder [3, 10]. The sustainable sourcing of MgO notwithstanding, the environmental footprint of cementitious products made from MgO/HY further depends on their long-term durability. For the particular case of structural concrete reinforced with steel, the corrosion performance of carbon steel embedded in concrete made with these novel binders is of paramount importance.

Chloride-induced corrosion is recognized as one of the major causes for the structural degradation of reinforced concrete structures [11]. In addition to the local breakdown of steel passivity [12, 13], chloride ingress into reinforced concrete further induces the formation of chloride phases, i.e. Kuzel's salt (C_3_A⋅0.5CaCl_2_⋅0.5CaSO_4_⋅10H_2_O) and Friedel's salt (C_3_A⋅CaCl_2_⋅10H_2_O) [14–16] in case of concrete based on conventional Portland cement. Whilst Mg-cements are generally deemed incompatible with steel reinforcement due their low pore solution pH [17], the formation of Mg-chlorides in MgO/HY concrete may be positive, in that it may limit the free chloride concentration at disposal to catalyze steel corrosion. However, to the best of our knowledge, no results about the durability of MgO/HY binder exposed to chloride-rich environments have been reported thus far in the literature.

In comparison to traditional Portland cements, the pore solution of MgO/HY binder has a low pH of approx. 10.5–11.0 [3]. From a durability viewpoint, this comparatively moderate degree of pore solution alkalinity is considered a pitfall, as the protective film of iron (hydr)oxide phases on the surface of the steel reinforcement ceases to be thermodynamically stable [18]. In contrast to the prevailing doctrine that the loss in reinforcement passivity inevitably leads to accelerated corrosion [19], a number of publications [20–23] as well as practical case studies [24] show that the moisture content is a more accurate predictor of the reinforcement corrosion rate. The question as to whether the pore solution pH of MgO/HY binder is, analogous to the corrosion of steel in carbonated Portland cement concrete, tolerable, as long as the moisture condition can be controlled, must be further investigated.

This paper investigates the durability of the MgO/HY binder with regard to its resistance against chloride attack, the ingress of moisture and corrosion performance. The resistance against chloride attack was investigated by rapid chloride ingress tests of MgO/HY mortars according to the Swiss standard SIA 262/1 Appendix B. A rapid chloride ingress test is a common method to assess the susceptibility of Portland cement-based reinforced concrete to chloride-induced corrosion. Phase analyses of pastes cured in alkaline chloride solution (the same solution as used for the chloride ingress tests) supplemented the mortar experiments to study the potential formation of Mg-chlorides. Linear polarization resistance (LPR) and single-frequency impedance measurements were done to provide further insights into the corrosion performance of the MgO/HY binder under the capillary uptake of (i) water and (ii) alkaline, concentrated chloride-containing solution. Findings are discussed with particular emphasis on the compatibility of the MgO/HY binder with steel reinforcement. Since mechanical properties of MgO/HY mortars have been shown to depend on the MgO-to-HY ratio and the curing environment [25], in particular the relative humidity (RH), two different MgO/HY mortar mixes with MgO-to-HY ratios of 90/10 and 70/30 by mass were prepared for the experiments and cured at high (98%) and rather low RH (57%). Mechanical tests of MgO/HY pastes and mortars in previous studies [2, 4] demonstrate that a high MgO-to-HY ratio is beneficial for strength. The only hydrate present in the MgO/HY binder is the HCB phase, whose formation is associated with a decrease in porosity, as water is bound within the phase. Under the constraint that the entire MgO can be converted to the HCB phase in presence of HY, a high MgO-to-HY ratio leads to the formation of a high amount of HCB, which results in lower porosity and ultimately in higher strength. Therefore, MgO/HY mortars with relatively high MgO contents, i.e. 90/10, and 70/30 MgO/HY by mass, were investigated in this study.

## Materials and methods

### Materials

The MgO/HY binder was mixed from commercially available raw materials. The reactive MgO powder was a calcined product of natural magnesite of medium reactivity with a specific surface area (SSA) of 31.1 m^2^/g measured by nitrogen gas adsorption (range of SSA of medium reactive MgO: 10–60 m^2^/g [26]). The HY was a natural mix of HY and huntite (CaMg_3_(CO_3_)_4_). The mineral composition of the natural mix was determined by Rietveld refinement, yielding 67% HY, 25% huntite, 7% dolomite (CaMg(CO_3_)_2_), and 1% other minor phases, e.g. quartz. A comparison of the chemical composition, the specific surface area and bulk density of both the rective MgO powder and the natural HY/huntite mix is enclosed in Table 1. Their X-ray diffraction patterns are appended to this study as electronic supplementary materials (compare Figures A1 and A2). The particle sizes were measured with a laser particle size analyzer (Malvern Mastersizer X, Germany), using isopropanol for dispersion (compare Figure A3). The binder raw materials were homogenized with a Turbula mixer in a 1 L plastic bottle for 2 h.

**Table 1:** Chemical composition, bulk density, and specific surface area (SSA) of binder raw materials

Oxides contents [wt%]^[a]^	Reactive MgO	Natural HY/huntite mix
SiO_2_	0.39	0.58
Al_2_O_3_	0.19	< 0.11
Fe_2_O_3_	0.65	0.05
Cr_2_O_3_	< 0.003	< 0.003
MnO	0.024	< 0.004
TiO_2_	< 0.019	< 0.019
P_2_O_5_	< 0.017	< 0.017
CaO	1.93	6.87
MgO	93.63	38.81
K_2_O	0.05	< 0.03
Na_2_O	< 0.06	< 0.06
SO_3_	0.56	0.08
*LOI*	2.52	53.48
sum	99.94	99.87
CO_2_ [wt%]^[b]^	4.8	40.4
bulk density [g/cm^3^]^[c]^	3.45	2.42
SSA [m^2^/g]^[d]^	31.1	10.0

[a] Oxide contents determined according to EN 196–2. *LOI* = loss on ignition

[b] Total carbon content was measured according to DIN ISO 10694 and used to calculate CO_2_ mass fraction

[c] Bulk density was determined by pycnometry according to EN 196–6

[d] Nitrogen gas adsorption measurements were performed to measure SSA using a Brunauer–Emmett–Teller (BET) model

**Fig. 1:**
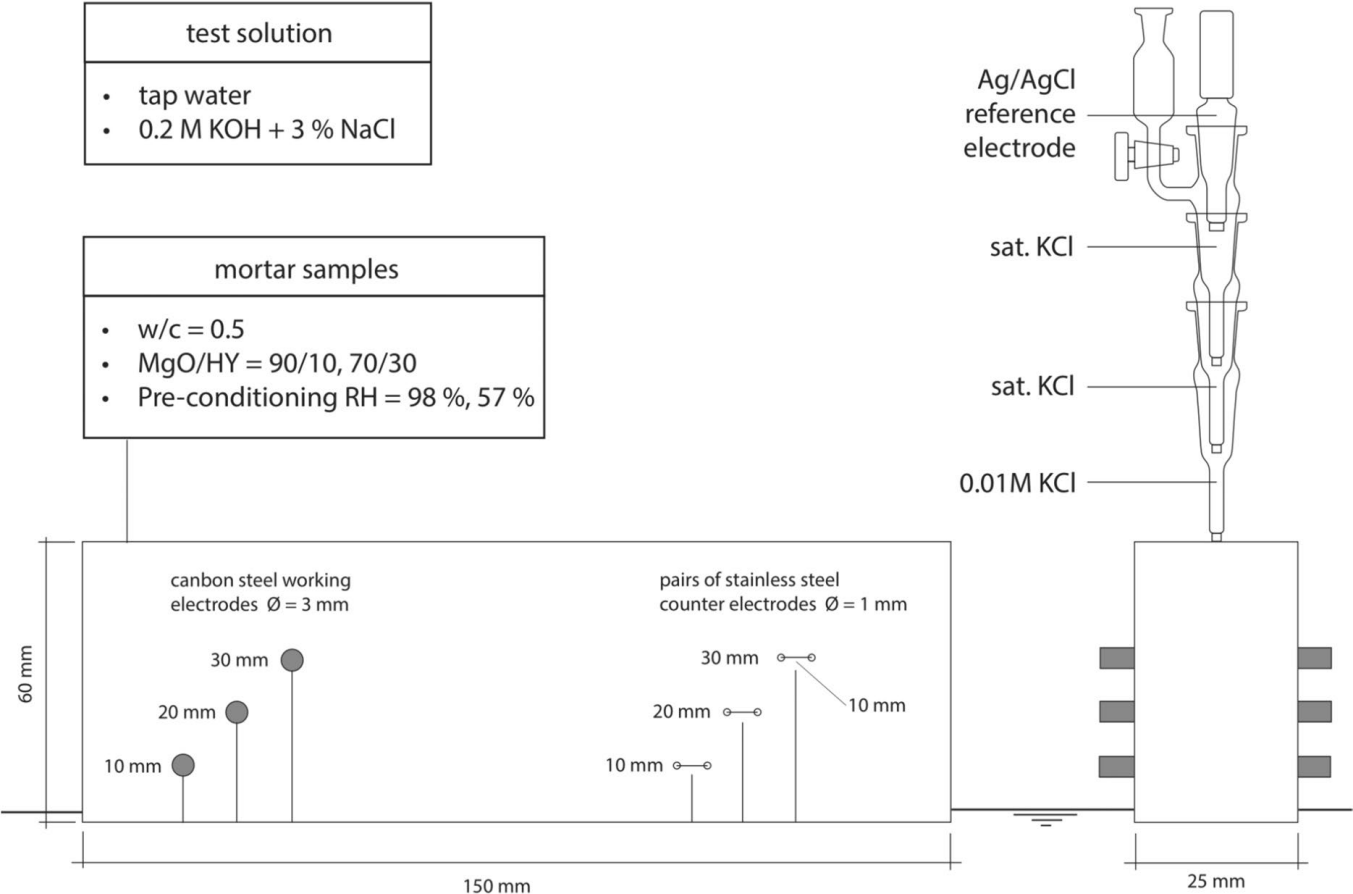
Schematic illustration of the electrochemical setup to monitor the corrosion rate of carbon steel in MgO/HY mortar samples exposed to the capillary ingress of (i) tap water and (ii) 0.2 M KOH + 3% NaCl

**Fig. 2:**
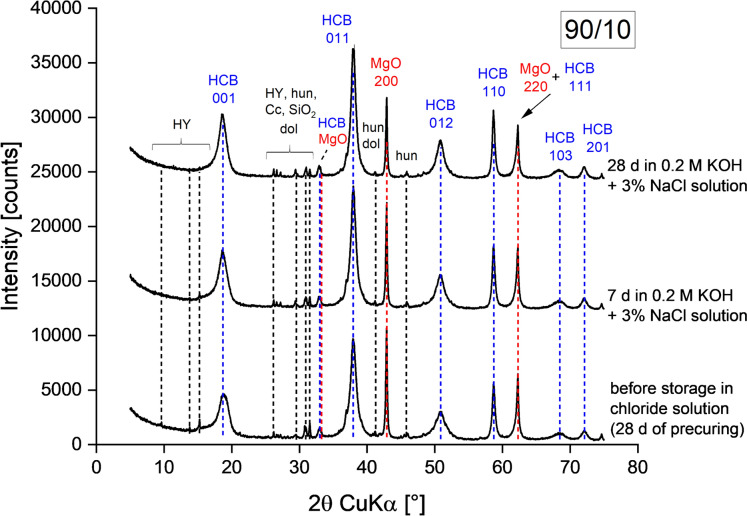
Diffraction patterns of a 90/10 paste cured at 98% RH for 28 d (reference before curing in alkaline chloride solution (0.2 M KOH + 3% NaCl)) and after curing in alkaline chloride solution for 7 d and 28 d. HCB: hydrous carbonate-containing brucite; HY: hydromagnesite; hun: huntite; dol: dolomite; Cc: calcite

**Fig. 3:**
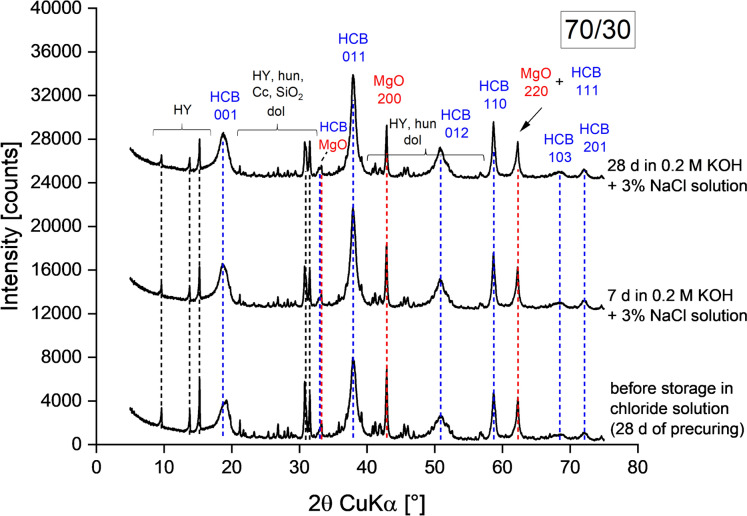
Diffraction patterns of a 70/30 paste cured at 98% RH for 28 d (reference before curing in alkaline chloride solution (0.2 M KOH + 3% NaCl)) and after curing in alkaline chloride solution for 7 d and 28 d. HCB: hydrous carbonate-containing brucite; HY: hydromagnesite; hun: huntite; dol: dolomite; Cc: calcite

MgO/HY pastes for phase analyses were prepared with a w/c of 0.50 using deionized water and the addition of 3.5% polycarboxylate ether (PCE) superplasticizer (solid content: 32%, dissolved in the mixing water). Pastes were mixed with a vacuum mixer and filled into cylindrical plastic vessels (inner diameter 33 mm), sealed and cured at 20 °C for 24 h. Mortars for corrosion experiments and rapid chloride ingress tests were prepared with a sand-to-binder ratio of 3:1 by mass, using an aggregate mixture (Normensand GmbH, Beckum, Germany) conforming to EN 196–1. The mortars were mixed according to the mixing procedure described in EN 196–1. After mixing, mortars were filled into molds and compacted by vibration. Mortars were cured at 20 °C and 98% RH for 24 h, demolded and transferred into their dedicated curing environments, as described in the following method sections.

### Methods

#### Phase analyses of pastes

Phase analyses were performed on 90/10 and 70/30 pastes. The pastes were mixed and cured for 24 h, after which the samples were taken out of the plastic vessels and cut into 5.0–5.5 mm-thick slices with an approximate mass of 7–8 g. Two 90/10 and 70/30 paste slices were kept as references and cured at 20 °C and 98% RH for another 27 d. The other 90/10 and 70/30 paste slices were pre-conditioned at 20 °C and 98% RH for 28 d (stage I) and then subsequently cured in 0.2 M KOH + 3% NaCl solution at 20 °C for 7 and 28 d (stage II) (see Table 2). To discriminate the effect of pH and chlorides, a separate set of pastes was cured in an alkaline solution (0.2 M KOH) without any NaCl addition to solely see the effect of the high pH on the hydrate assemblage. After curing, paste slices were crushed and hydration was stopped by organic solvent exchange [24]. The pastes were then ground below 0.063 mm and stored until XRD and TGA measurements in plastic bags in a desiccator under vacuum.

**Table 2:** MgO/HY pastes prepared for phase analysis. All samples were prepared with a w/c ratio of 0.50 and 3.5% SP concentration

	Stage I, pre-conditioning		Stage II, curing	
MgO-to-HY mixing ratio	Relative humidity	Curing time	solution	Curing time
90/10 (reference)	98%	28 d	–	–
90/10	98%	28 d	0.2 M KOH	7 d, 28 d
90/10	98%	28 d	0.2 M KOH + 3% NaCl	7 d, 28 d
70/30 (reference)	98%	28 d	–	–
70/30	98%	28 d	0.2 M KOH	7 d, 28 d
70/30	98%	28 d	0.2 M KOH + 3% NaCl	7 d, 28 d

X-ray diffraction patterns were measured with an X'Pert Pro (Malvern Panalytical, UK) in Bragg–Brentano geometry. The diffractometer uses a scanning line detector X'Celerator (Malvern Panalytical, UK), Cu Kα_1_ radiation (λ = 1.54059 Å) and a Ge (111) Johansson monochromator. All samples were scanned across a range of 2θ = 5 – 75˚ in steps of 0.0167° 2θ.

Ground paste powders were analyzed by thermogravimetry using an STA 449 F3 Jupiter TGA instrument (Netzsch, Germany). Approximately 50 to 60 mg of the powders were filled into an alumina crucible (Al_2_O_3_, mass = 325 mg) and heated under a nitrogen atmosphere from 30 to 1000˚C at a heating rate of 10 K/min. The nitrogen gas flow rate was 20 ml/min.

#### Rapid chloride ingress test on mortar samples

The rapid chloride ingress test was performed according to the Swiss standard SIA 262/1 Appendix B, which specifies that a Portland cement-based concrete of certain exposure classes must pass an accelerated chloride ingress test. Chloride attack resistance is assessed by the chloride migration coefficient ${D}_{Cl}$, which must not exceed 10 · 10^–12^ m^2^/s for exposure classes XD2b(CH) and XD3(CH) to pass the test. The rapid chloride ingress test as described in the standard was chosen as a comparably quick and simple test to assess the MgO/HY binder's chloride penetration resistance enabling to compare the binder's durability to Portland cement-based binders in chloride-rich environment. However, the experiments were conducted under the following two constraints: (i) the specimens were mortars and not concrete samples, and (ii) the pH of the chloride test solution is specified in the standard to match the pH of the pore solution in Portland cement (pH = 13.3) and is therefore higher than the pH of the pore solution of the MgO/HY binder (pH = 10.5–11.0) [3].

90/10 and 70/30 MgO/HY mortars for rapid chloride ingress tests were filled in 150 $\times$ 150 $\times$ 150 mm^3^ cube molds. The cube molds were demolded after 24 h of curing at 98% RH and then cured for another 19 d. After a total curing time of 20 d, three cylindrical samples of 50 mm diameter and length were drilled from the cubes. Subsequently, the cylindrical samples were cured for another 7 d in deionized water and then prepared for testing (after 28 d in total). Before the measurement, the cores were cleaned in an ultrasonic bath for 120 s and dried with a cloth. Then, a latex foil was sealed around the cylindrical samples with the original surface and its opposite surface remaining uncovered by the foil. The samples were placed into separate measurement devices, which were cured in alkaline chloride solution (0.2 M KOH + 3% NaCl).

The measurement device consisted of a metal cage, with an anode and a cathode linked to a power source (DC) and a cylinder filled with a chloride-free, alkaline solution (0.2 M KOH) (compare Figure A4). The two opposing, uncovered sides of the cylindrical samples were brought into contact with the chloride-free, alkaline solution (side of the anode) and the alkaline chloride solution (side of the cathode). The measurements were started by applying 20 V to induce chloride migration towards the anode. Current, voltage, and temperature of both solutions were monitored and, if necessary, adjusted during the measurements. After 24 h the measurement was stopped and the samples were taken out, dried and split in halves to examine the longitudinal cross-section. To reveal chloride migration fronts, cross-sections of samples were dyed with 0.1% fluorescein solution (ethanol as solvent) and 0.1 M silver nitrate solution. Areas into which the chlorides had penetrated were coloured pink by the dyeing procedure. The samples were then dried at 40 °C for another 24 h. After drying, the pink colour in chloride-rich areas was bleached out, while areas without significant chloride concentrations appeared much darker. Chloride penetration depth was measured at six equidistant points for each cross-section of a sample. The depth was measured from the sample's surface, which was in contact with the chloride solution, to the chloride migration front. Wet bulk density ${\rho }_{wet}$ and chloride migration coefficient *D*_*Cl*_ were determined using Eq. 1 and Eq. 2, respectively.

**Fig. 4:**
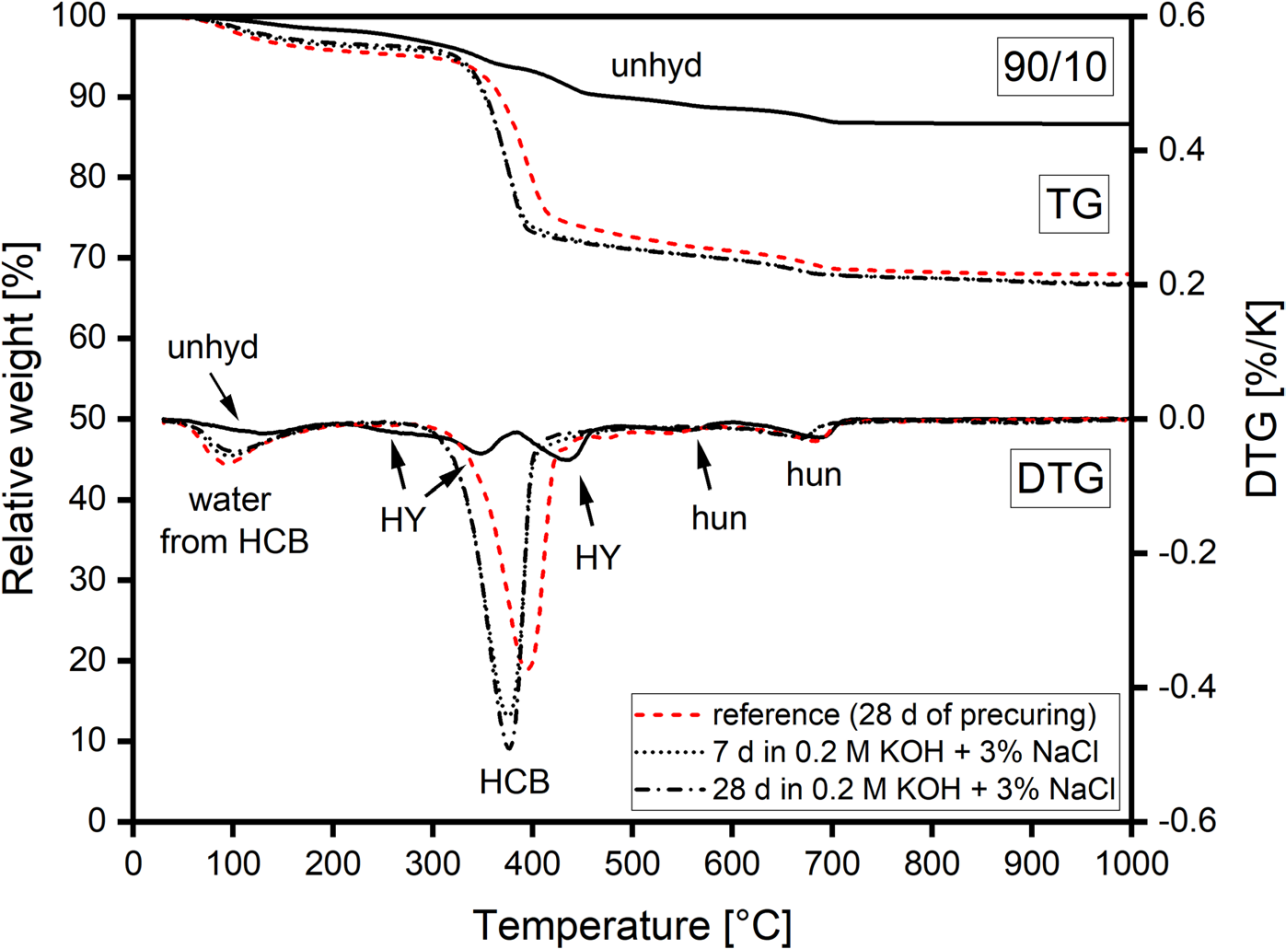
TGA data of an unhydrated 90/10 binder and a 90/10 paste cured at 98% RH for 28 d (reference before curing in alkaline chloride solution (0.2 M KOH + 3% NaCl)) and after curing in alkaline chloride solution for 7 d and 28 d. unhyd: unhydrated binder; HCB: hydrous carbonate-containing brucite; HY: hydromagnesite; hun: huntite

1 $${\rho }_{wet}= \frac{4{m}_{w}}{{d}^{2}\pi h}$$

with ${m}_{w}$ = sample mass after storage in water [kg], $d$ = sample diameter [m], $h$ = sample height [m].

2 $$D_{{{\text{Cl}}}} = \frac{z}{t} \cdot \left( {x_{d} - 1.5462} \right) \cdot \sqrt {z \cdot x_{d} } \cdot [m^{2} /s]$$

with $z$ = 8.619 · 10^–5^ $\frac{hT}{U}$ [$m$] (*T* being average temperature of the alkaline chloride-free and chloride-rich solutions [K], $U$ = applied voltage [V]), $t$ = measuring time [s], ${x}_{d}$ = mean chloride ingress depth [m].

#### Electrochemical measurements on mortar samples

The corrosion performance of carbon steel in 90/10 and 70/30 mortars was studied using an experimental setup shown in Fig. 1. The specimen design and the placement of embedded working and counter electrodes was further optimized to allow for the simultaneous monitoring of moisture ingress through capillary suction, as detailed in Schmid et al. [27]. As schematically illustrated in Fig. 1, carbon steel rods and pairs of stainless steel rods were embedded in mortar prisms (150 × 25 × 60 mm^3^) at a cover depth of 10, 20 and 30 mm relative to the sample base. Previous to the electrochemical measurements, 90/10 and 70/30 mortars were pre-conditioned for 28 days at 20 °C and 98% or 57% RH to investigate the effect of the curing condition on the corrosion performance of the binder as well as its resistance to the ingress of moisture. The prisms were placed in a test solution reservoir of (i) tap water and (ii) 0.2 M KOH + 3% NaCl solution. The ingress of electrolyte perpendicular to the water table was monitored in a series of single-frequency two-point impedance measurements between the pairs of equidistant stainless steel rods. One mortar prism was used for each test solution and curing condition. Impedance measurements were performed continually for 3 to 5 days in a 5-min interval, at a frequency of 1000 Hz and an AC amplitude of 1 V, using a data logger and sensor system provided by the company DuraMon (Zürich, Switzerland).

The instantaneous corrosion rate of carbon steel embedded in MgO/HY mortars was further evaluated by a series of linear polarization resistance (LPR) measurements in a 3-electrode setup. For each embedded carbon steel working electrode (WE), the stainless steel rod closest to the WE at the same cover depth was used as counter electrode (CE). Working electrodes underwent potentiodynamic polarization from −20 to + 20 mV relative to the open circuit potential (OCP), and then back down again to −20 mV in cathodic direction at a scan rate of ν = 0.167 mV/s. The instantaneous current density, ${i}_{\text{corr}}$, was computed according to.

3 $${i}_{\text{corr}}= \frac{{b}_{a}\times {b}_{c}}{{\text{ln}}\left(10\right)\times ({b}_{a}+{b}_{c})}\times \frac{1}{{R}_{p}} [\text{A}/{\text{cm}}^{2}],$$

where ${R}_{p}$ is the polarization resistance in $\Omega {\text{cm}}^{2}$ and ${b}_{a}$ and ${b}_{c}$ are the anodic and cathodic Tafel slopes in V [27], where $\frac{{b}_{a}\times {b}_{c}}{{b}_{a}+{b}_{c}}=12 {\text{mV}}$.

All measurements used a silver-silver chloride (Ag/AgCl_sat_) reference electrode (RE) in saturated KCl manufactured by Willi Möller AG (Zürich, Switzerland). Before each experiment, the RE potential was calibrated and the measured potentials corrected against a laboratory-internal reference-reference electrode. The RE was assembled into a stack of three porous frit glass capillaries, the two topmost capillaries containing saturated KCl and the bottom one containing diluted 0.01 M KCl, to ensure a stable reference potential and prevent the leakage of highly concentrated chloride solution into the mortar sample. The RE stack was placed on top of the mortar prisms, as illustrated in Fig. 1.

## Results

### Phase analyses of pastes cured in chloride solution

#### XRD

Figures 2 and 3 show diffraction patterns of 90/10 and 70/30 pastes before and after curing in alkaline chloride solution, respectively. Pre-curing of pastes at 20 °C and 98% RH for 28 d led to hydration of MgO and HY resulting in the formation of the HCB phase, the main hydration product in MgO/HY blends [3, 25]. The reflections of the HCB phase showed characteristic broadening and 001 reflection splitting as a result of stacking faults [10].

The formation of Mg-chlorides or any other additional phases besides the HCB phase was not observed in 90/10 and 70/30 pastes cured for 7 and 28 d in alkaline chloride solution. Thus, the presence of chlorides in solution did not affect the general phase assemblage of the MgO/HY pastes. Curing in alkaline chloride solution led only to further hydration of MgO and HY, resulting in formation of more HCB. Figures A5 and A6 in the ESM compare 90/10 and 70/30 pastes cured in alkaline solution (0.2 M KOH) and alkaline chloride solution (0.2 M KOH + 3% NaCl). The pastes did not show any significant difference with regard to phases found across all binder compositions and exposure conditions.

**Fig. 5:**
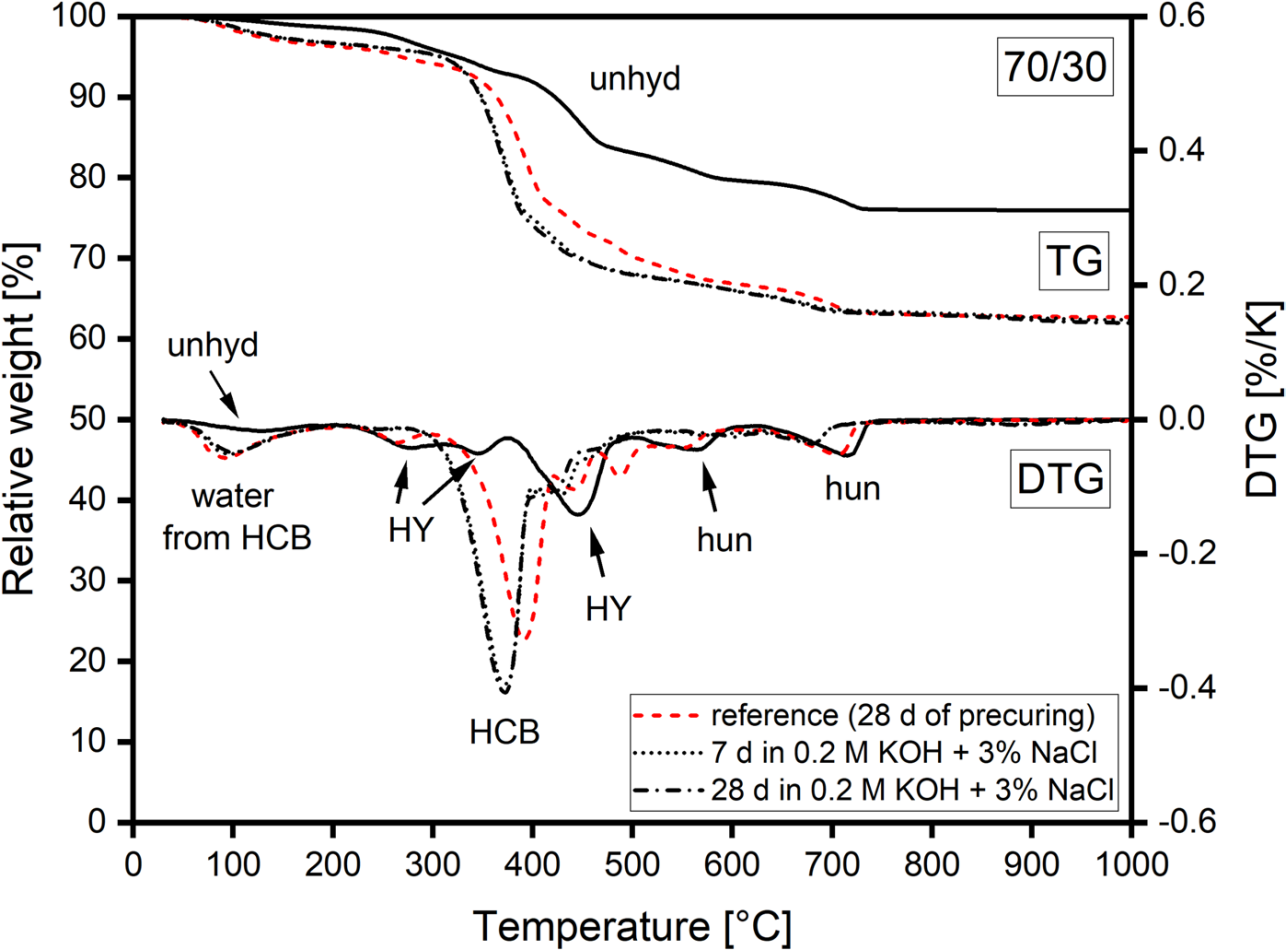
TGA data of an unhydrated 70/30 binder and a 70/30 paste cured at 98% RH for 28 d (reference before curing in alkaline chloride solution (0.2 M KOH + 3% NaCl)) and after curing in alkaline chloride solution for 7 d and 28 d. unhyd: unhydrated binder; HCB: hydrous carbonate-containing brucite; HY: hydromagnesite; hun: huntite

**Fig. 6:**
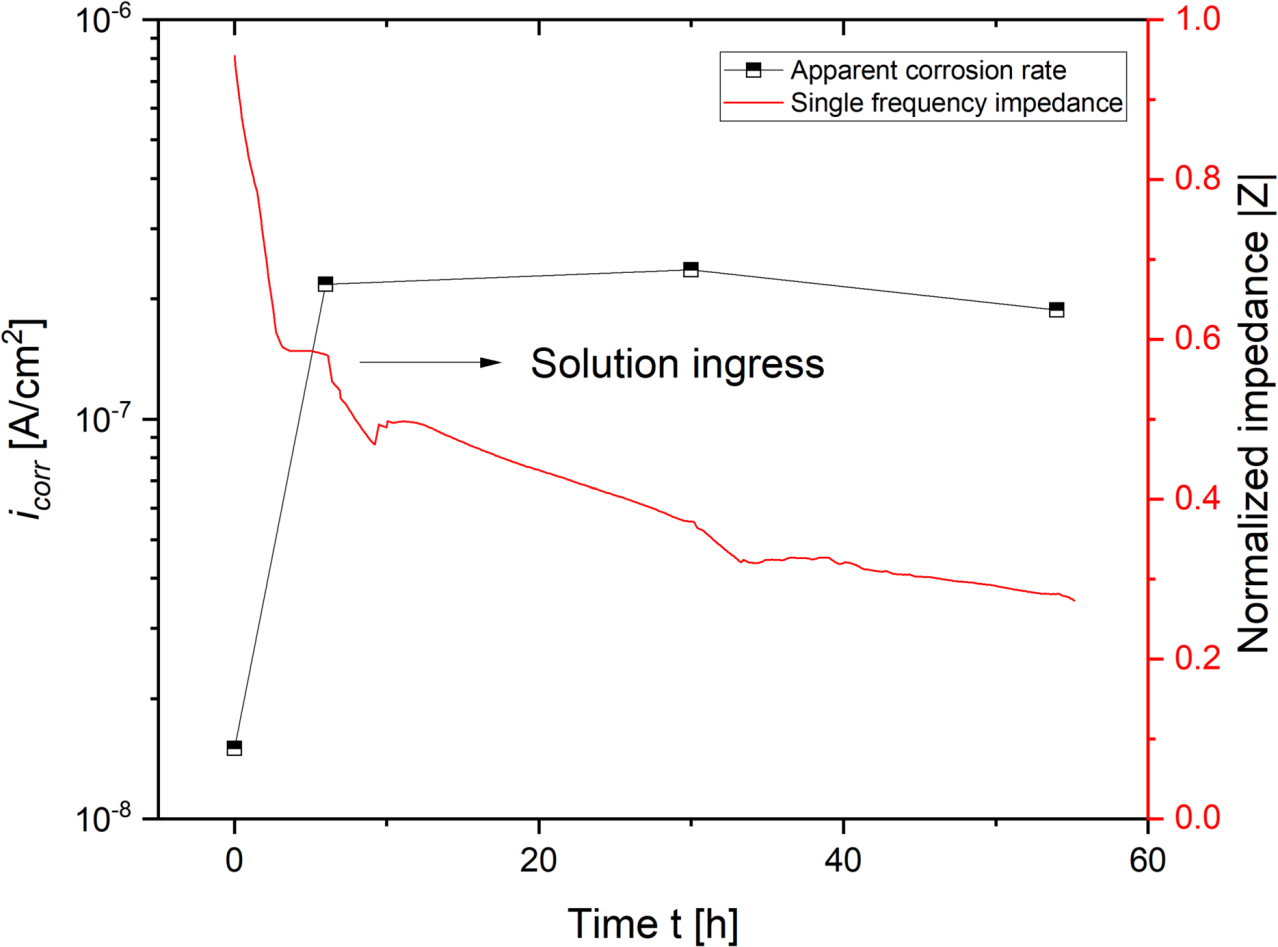
Corrosion current densities of carbon steel rods embedded in 90/10 samples cured at 98% exposed to the ingress of alkaline chloride-containing solution, together with the normalized single-frequency two-point impedance measurements between the stainless steel rods embedded at a cover depth of 10 mm, relative to the sample base

#### TGA

Figures 4 and 5 show TGA results of 90/10 and 70/30 reference pastes after pre-conditioning and pastes cured for further 7 and 28 d in alkaline chloride solution. Formation of the HCB phase was evident in both reference pastes from the high mass loss between 300 and 430 °C linked mainly to the loss of H_2_O and the release of CO_2_ from the decomposition of carbonate incorporated by the HCB phase [3]. The mass loss of the HCB phase in the 70/30 reference was lower due to the smaller amount of HCB formed. In addition, a decomposition peak of unreacted HY was measured at around 440 °C in the 70/30 reference. Minor decomposition peaks of huntite (stepwise decarbonation) were measured at around 560 °C and between 630 and 700 °C (630–730 °C for the 70/30 reference). Mass loss due to water from the HCB phase was observed between 30 and 200 °C for both reference pastes.

Pastes cured for 7 and 28 d in alkaline chloride solution did not show any additional, separate decomposition peak related to newly formed phases, e.g. Mg-chlorides. However, such peaks, if present, could be masked by the larger decomposition peak of the HCB phase or by unreacted HY or huntite.

The mass loss related to decomposition of HCB between 300 and 430 °C was higher for pastes cured in alkaline chloride solution. This correlates with the observations from XRD, where advancing hydration led to higher HCB content in samples. However, curing in alkaline chloride solution resulted in a strong shift of the main HCB decomposition peak towards lower temperatures. A comparison of TGA data of pastes cured in the alkaline chloride-free solution and in the alkaline chloride solution (Figures A7 and A8 in the ESM) revealed that the shift of the decomposition peak of HCB was the result of chloride additions to the curing solution. While reference pastes and pastes cured in alkaline solution showed no shift of the HCB peak, pastes cured in alkaline chloride solution exhibited a significant shift. Furthermore, curing in solutions with and without chlorides had an effect on the amount of water, which was released during the TGA measurement between 30 and 200 °C. Pastes cured in alkaline chloride solution exhibited in general lower amounts of water than the reference pastes.

**Fig. 7:**
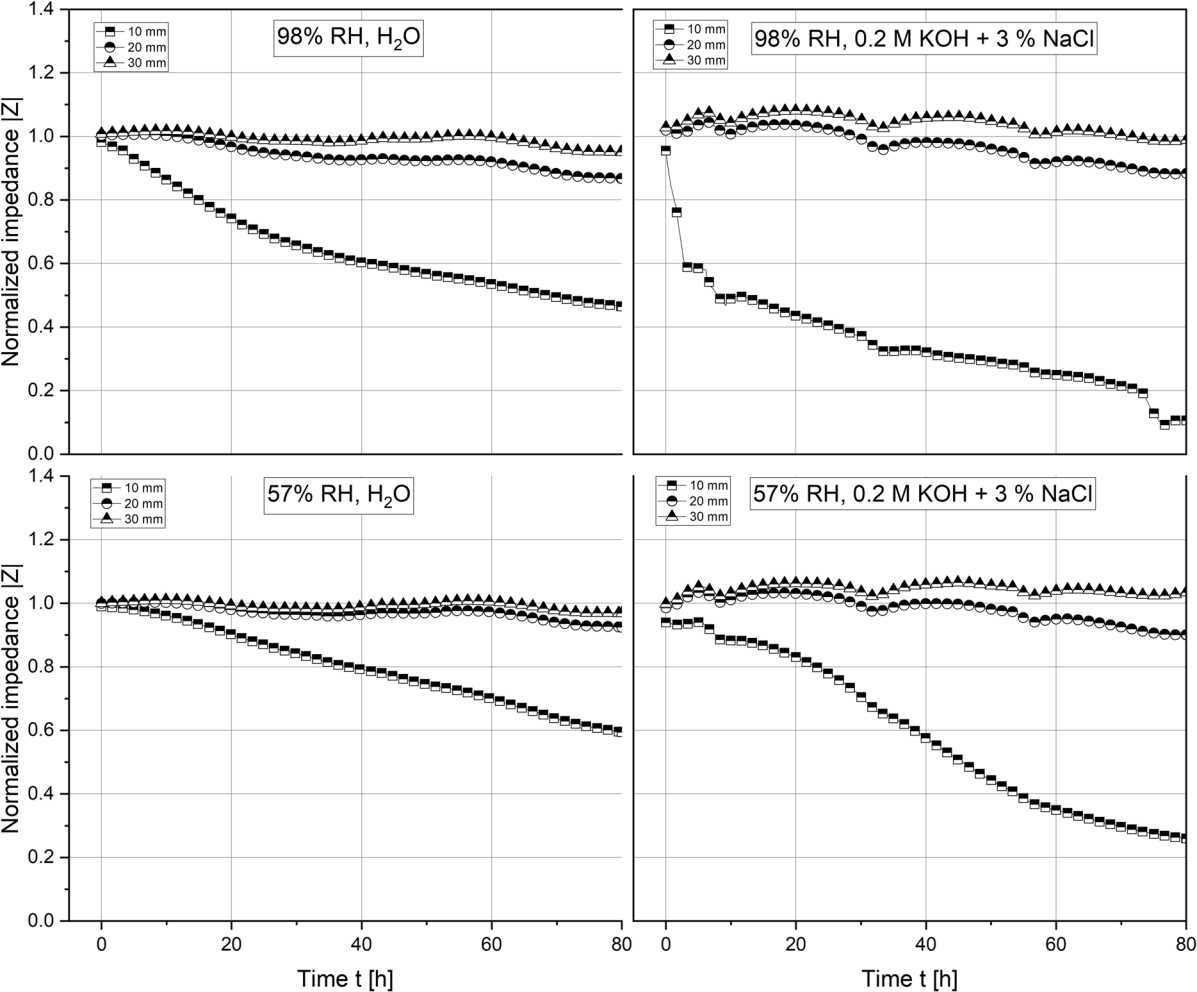
Normalized single-frequency two-point impedance between the stainless steel rods embedded in 90/10 samples cured at 98% and 57% RH as a function of the exposure time and cover depth, relative to the sample base

**Fig. 8:**
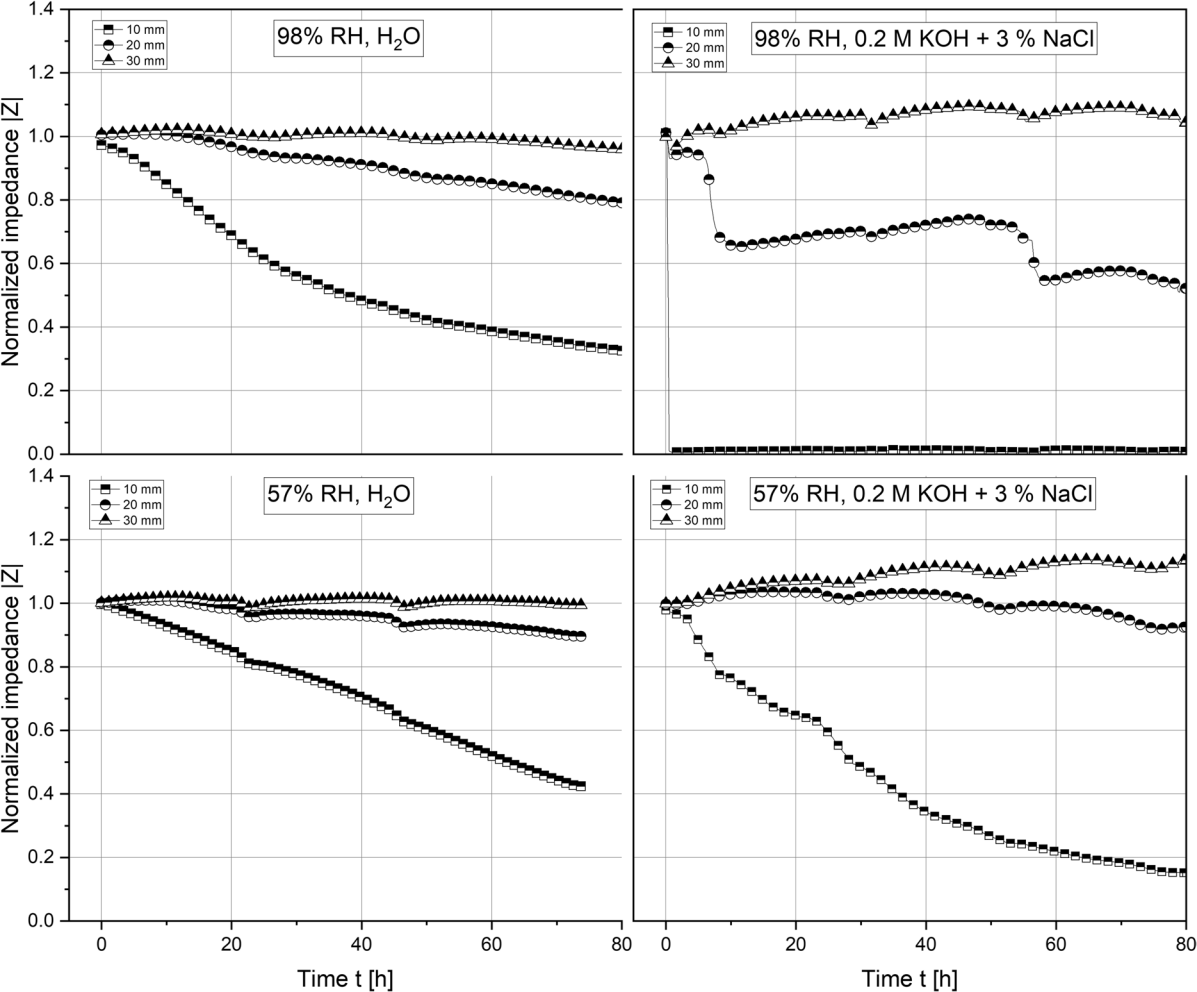
Normalized single-frequency two-point impedance between the stainless steel rods embedded in 90/10 samples cured at 98% and 57% RH as a function of the exposure time and cover depth, relative to the sample base

### Rapid chloride ingress test

Mortars of both binder compositions showed only marginal chloride penetration depths between 1 and 5 mm (total sample length: 50 mm), with mean penetration depths of around 2–3 mm (see Tables 3 and 4 for 90/10 and 70/30 MgO/HY mortars, respectively). Graphical representations of chloride penetration profiles for 90/10 and 70/30 mortars are displayed in Figures A9 and A10 in the ESM, respectively. Figure A11 in the ESM shows exemplary pictures of 90/10 mortar halves, split and dyed. Mean chloride migration coefficients *D*_*Cl*_ were similar for both samples: $(0.6\pm 0.3)\times {10}^{-12}$ and $\left(1.0 \pm 0.6\right)\times {10}^{-12}$ m^2^/s for the 90/10 and 70/30 mortars, respectively.

**Table 3:** Chloride ingress test results of the 90/10 mortar

	Sample #1	Sample #2	Sample #3	Mean
Diameter [mm]	49.5	49.6	49.5	49.5 ± 0.0
Wet bulk density [kg/m^3^]	2199	2190	2202	2197 ± 7.0
Applied voltage [V]	20.3	20.4	20.3	20.3 ± 0.1
Mean chloride ingress depth [mm]	1.3	0.8	1.9	1.3 ± 0.6
Max. chloride ingress depth [mm]	2	3	4	3.0 ± 1.0
Chloride migration coefficient D_Cl_ [m^2^/s]	${0.6\times 10}^{-12}$	${0.3\times 10}^{-12}$	${1.0\times 10}^{-12}$	$(0.6\pm 0.3)\times {10}^{-12}$

**Table 4:** Chloride ingress test results of the 70/30 mortar

	Sample #1	Sample #2		Sample #3	Mean
Diameter [mm]	49.5		49.4	49.5	49.5 ± 0.1
Wet bulk density [kg/m^3^]	1972		1992	1968	1977 ± 13.0
Applied voltage [V]	20.0		20.2	20.2	20.2 ± 0.0
Mean chloride ingress depth [mm]	3.1		1.2	1.7	2.0 ± 1.0
Max. chloride ingress depth [mm]	5		2	4	3.7 ± 1.5
Chloride migration coefficient D_Cl_ [m^2^/s]	${1.7\times 10}^{-12}$		${0.5\times 10}^{-12}$	${0.8\times 10}^{-12}$	$\left(1.0 \pm 0.6\right)\times {10}^{-12}$

**Fig. 9:**
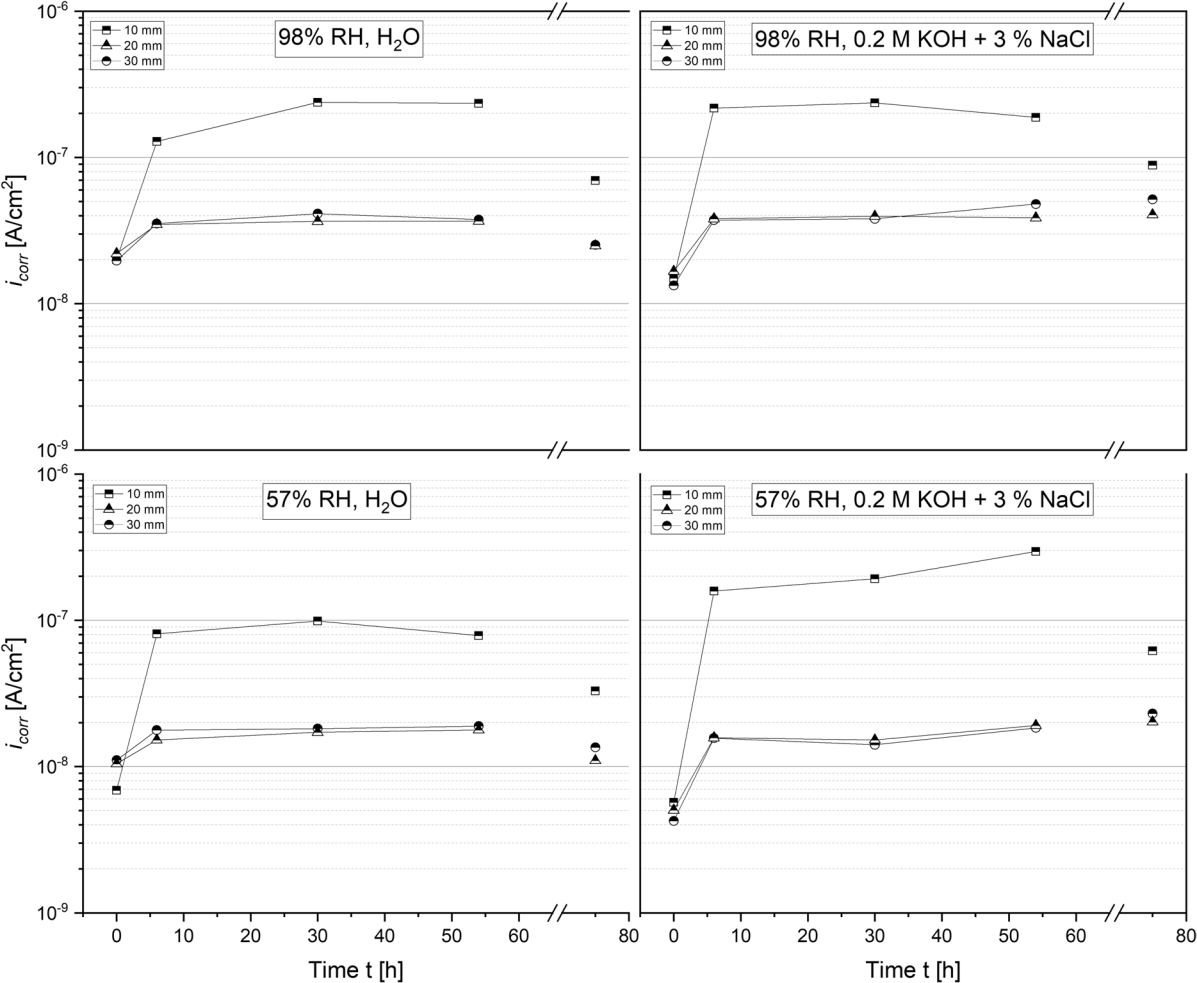
Corrosion current densities of carbon steel rods embedded in 90/10 samples cured at 98% and 57% RH as a function of the exposure time and cover depth, relative to the sample base

**Fig. 10:**
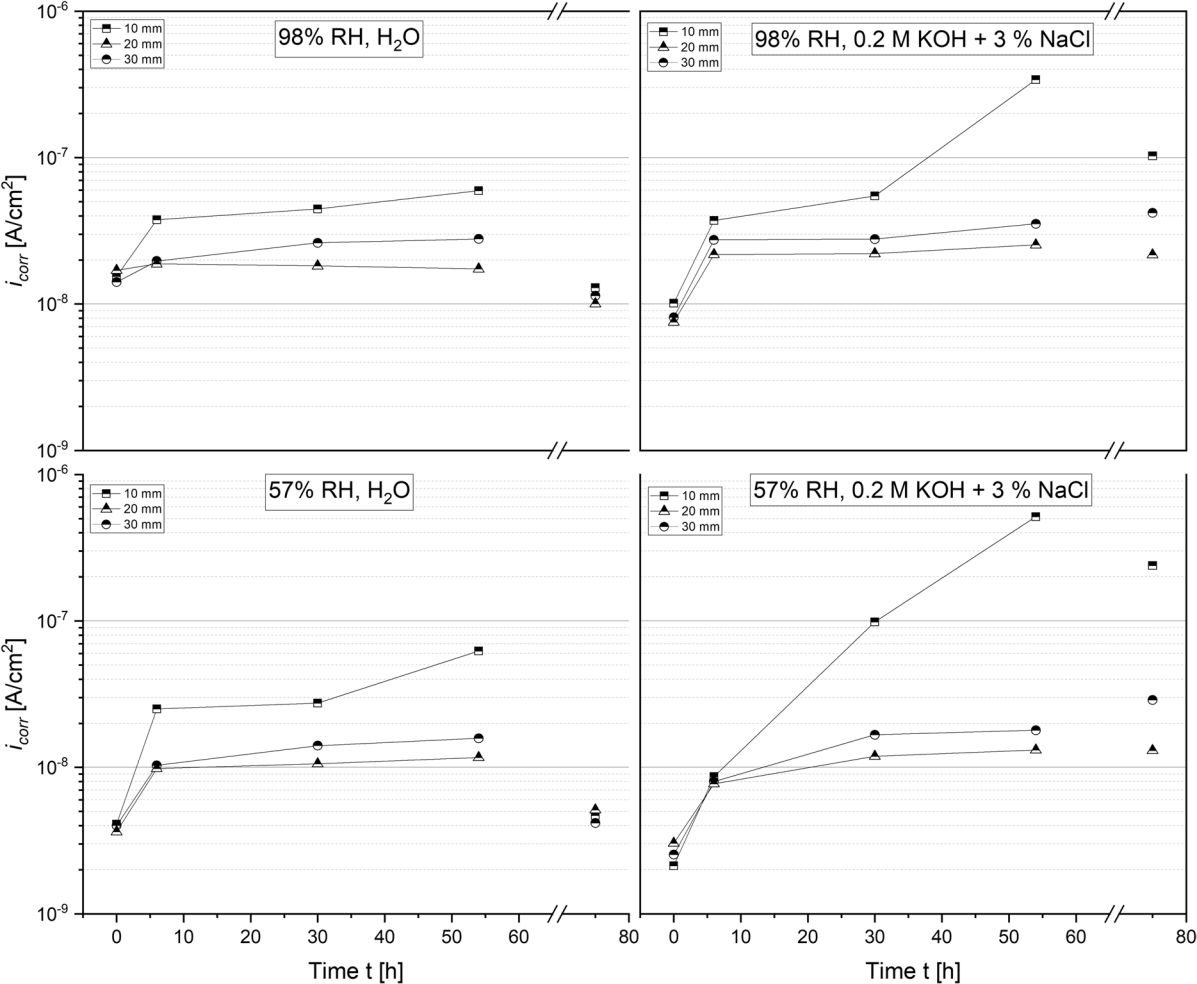
Corrosion current densities of carbon steel rods embedded in 70/30 samples cured at 98% and 57% RH as a function of the exposure time and cover depth, relative to the sample base

**Fig. 11:**
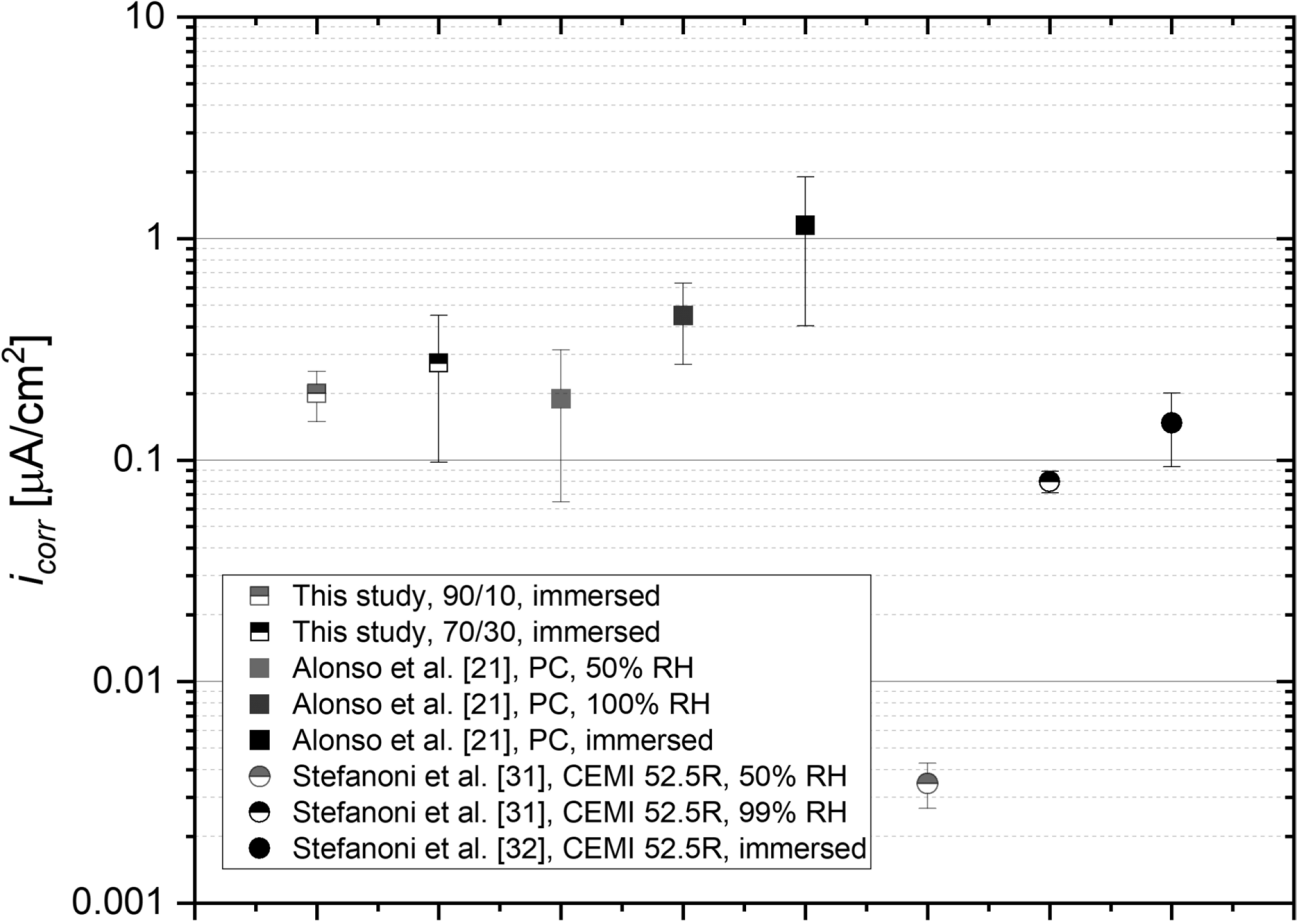
The average corrosion rate of carbon steel, embedded in 90/10 and 70/30 blends of MgO/HY mortars (w/c = 0.50, cover depth = 10 mm) exposed to the ingress of water, together with the average ${i}_{corr}$ measured in carbonated Portland cement [21] and CEMI [31, 32] systems of comparable w/c

### Corrosion experiments of steel rebars embedded in MgO/HY mortars

Transient electrochemical measurements performed show that the ingress of test solution across the height of the sample coincides with a reduction in single-frequency impedance measurements between the pairs of stainless steel counter electrodes and an increase in the apparent corrosion rate of carbon steel rods embedded. The progression of both parameters across is schematically illustrated in Fig. 6.

Figures 7, 8 show various two-point impedance measurements as a function of the sample composition, exposure time, cover depth and curing conditions. Across all data series obtained, the normalized impedance measurements reduced the fastest for the pair of stainless steel rods closest to the water table, that is, at a cover depth of 10 mm, followed by those embedded at 20 and 30 mm. 90/10 mortars responded equally rapid to the ingress of water, irrespective of the curing condition. Due to the high electrolyte conductivity, the ingress of highly concentrated chloride-containing solution prompted a more significant drop in the measured impedances than the ingress of tap water. At a cover depth of 30 mm, the observed reduction in the measured impedance appears insignificant across the entire time span of the experiment. 70/30 mortars were significantly less resistant to the capillary ingress of water, as evident from a faster and more pronounced reduction in the measured impedances compared to the 90/10 mortars.

The observed decrease was close to instantaneous for samples cured at 98% RH, reaching values of 40% and 2%, respectively, relative to the initial single-frequency impedance within 2 h. Samples cured at 57% RH approached the same relative impedance, but the recorded reduction was significantly more gradual. Analogous to the 90/10 samples, steel rods embedded at a cover depth of 30 mm appeared unaffected by the capillary uptake of both tap water and concentrated chloride solution. At the intermediate cover depth of 20 mm, the reduction was more pronounced for the 70/30 samples than for the 90/10 ones in similar exposure and curing conditions. Note that the periodic disturbances in the measured single frequency impedances are due to disconnecting the setup for the linear polarization resistance measurements in a 24 h interval.

As shown in Figs. 9, 10, the corrosion current densities of carbon steel embedded MgO/HY blends were generally low and within the orders of $1\times {10}^{-9}$ to $1\times {10}^{-7}$ $\text{A/}{{\text{c}}{\text{m}}}^{2}$. Irrespective of the curing and exposure conditions, the instantaneous corrosion current density of the carbon steel rods embedded in the 90/10 mortar blend plateaued at $(2.0+0.5)\times {10}^{-8}$ $\text{A/}{\text{cm}}^{2}$ after 2 to 3 days at a cover depth of both 20 and 30 mm. Analogous to the ingress of moisture, the initial increase in ${i}_{corr}$ was more pronounced for the rebars closest to the water level. Prisms cured at 98% RH reached a plateauing current density of $(2.5\pm 0.5)\times {10}^{-7}$ $\text{A/}{\text{cm}}^{2}$ for the tap water and alkaline, chloride containing test solution (Fig. 9).

Samples cured at 57% RH featured a slightly lower corrosion rate of $(9.0\pm 1.0)\times {10}^{-8}$ $\text{A/}{\text{cm}}^{2}$ under the ingress of water at 10 mm. Upon removing the sample from the test solution, the corrosion rates decreased by a factor of 2 for all rebars exposed to the ingress of water as well as the carbon steel rods exposed to chloride-containing solution at the lowest cover depth. As elucidated in the description of the two-point impedance results, 70/30 mortars appeared to absorb chloride-containing solution more readily than the 90/10 mortars. Correspondingly, the measured corrosion current densities were the highest for both of the 70/30 mortars immersed in 0.2 M KOH + 3% NaCl. As illustrated in Fig. 10, the maximum instantaneous corrosion rate measured for the 98% and 57% RH sample amounted to $4\times {10}^{-7}$ $\text{A/}{\text{cm}}^{2}$ and $5\times {10}^{-7}$ $\text{A/}{\text{cm}}^{2}$ after 3 days, respectively. Even though the single-frequency impedance measurements suggest an equally rapid uptake of water into the 70/30 mortars (Fig. 8), the measured corrosion rates (Fig. 10) fell short of those recorded in the 90/10 mortars.

Apart from the observed order of magnitude increase in various ${i}_{corr}$ at the lowest cover depth (10 mm) compared to the remaining cover depths (20 and 30 mm) upon exposure to water, no clear trend can be established with respect to the sample composition, test solution or curing condition. It must be noted that the observed corrosion rates approach the lower limit at which the LPR method can reliably quantify instantaneous corrosion rate (0.1 µA/cm^2^ or less) [28]. For this reason, we do not consider that the differences in the measured ${i}_{corr}$ across various sample compositions and curing conditions at such low levels are significant.

## Discussion

### Phase analysis of pastes

Formation of Mg-chlorides was not observed for 90/10 or 70/30 pastes cured in an alkaline chloride solution for 28 d by XRD or TGA. Whether the high pH impeded the formation of Mg-chloride phases or the chosen experimental conditions such as chloride concentration, curing time or penetration depth were insufficient, cannot be deduced from the experiments. Nonetheless, some minor changes to the HCB phase have been observed by TGA experiments. The presence of chlorides led to a shift of the HCB decomposition peak between 300 and 430 °C towards lower temperatures. In the alkaline chloride-free solution the thermal decomposition behavior of the HCB phase remained unaffected. A shift of the decomposition peak could be the result of a change in chemical composition, for example, a possible uptake of chlorides by the HCB phase or a change in crystallite size. However, the latter would require notable chloride intake, which would have been noticed by XRD.

Besides the shift of the HCB peak, the change of the amount of water related to the HCB phase and observed by TGA at low temperatures was an unexpected result. The decrease of this water was observed for 90/10 and 70/30 pastes cured both in alkaline solution free of chlorides and in alkaline chloride solution. The decrease was not linked to the shift of the HCB's decomposition peak, since the shift of the peak was observed for pastes only cured in alkaline chloride solution. A possible explanation for the loss of water could be the high pH of both curing solutions, which could affect the carbonate-bicarbonate equilibrium and by this also the carbonate incorporated into HCB during pre-curing. This could affect the interlayers in a way that less water can be bound. The decrease of water could therefore indicate a transformation of some of the HCB to brucite, resulting in the release of carbonate and water.

### Rapid chloride ingress test

The rapid chloride ingress test performed on 90/10 and 70/30 mortars after SIA 262/1 Appendix B showed a high penetration resistance against chlorides. Both mortar mixes showed comparable chloride migration coefficients. However, the small penetration depths obtained by the rapid chloride ingress tests do not necessarily provide information on the potential of corrosion of rebars in a hypothetical steel-reinforced MgO/HY concrete. The following differences to tests on Portland cement-based concrete are needed to be accounted when interpreting the results from the chloride ingress tests in this study: (i) the standard SIA 262/1 Appendix B has been initially developed for concrete, a material which has a lower paste volume than the MgO/HY mortars used for the experiments. (ii) The standard has been designed to link chloride penetration depths to risk of corrosion in Portland cement-based concrete. Due to the testing of a novel binder, it cannot be guaranteed that the relationship used for Portland cement between chloride penetration depth and corrosion probability will remain valid. (iii) The SIA 262/1 Appendix B standard has been furthermore designed for PC-based binders exhibiting a high alkalinity of pore solution due to presence of portlandite. The pH of the two alkaline solutions (with and without NaCl addition) brought into contact with the MgO/HY mortars during the tests, does not match the low pH of the MgO/HY binder's pore solution. The impact of pH on the test results remains uninvestigated in the experiments. Overall, the rapid chloride ingress tests confirm a low permeability for the analyzed MgO/HY mortar mixes preventing fast chloride penetration but do not necessarily provide information on corrosion resistance of steel in the binder. With regard to future investigations into the chloride permeability of Mg-based and other low pH binders, it may thus be worthwhile considering modifying the standardized rapid chloride ingress test and adjusting the test solution pH to the prevailing degree of alkalinity in the binder investigated. As the accelerated ingress test conducted in this study is further not representative of the ingress of chlorides under natural conditions, it is recommended that the here presented preliminary investigation is complemented by other tests that emulate the ingress of chlorides due to bulk diffusion and advection, e.g. in cyclic wet/dry exposure.

### Corrosion experiments

The electrochemical measurements presented in this study further demonstrate a good short-term corrosion performance of both mortar compositions studied. Single-frequency impedance measurements show that the capillary ingress of water and chloride-containing test solution occurs more readily in the 70/30 mortar samples compared to the 90/10 mortars. These differences likely relate to differences in the porosity of both mortar mixes. As evident from a significant reduction in the normalized impedance measurements over time, the pore network in the vicinity of the steel rods embedded at a cover depth of 10 mm has become increasingly more saturated over the course of the experiment. Despite the continued ingress of moisture, their measured instantaneous corrosion rates do not exceed $5.0\times {10}^{-7}$ $\text{A/}{\text{cm}}^{2}$ (compare Figs. 9 and 10, 54 h), equivalent to a cross-sectional loss of less than $6$ µm/year. As elucidated in the previous section, various other corrosion rates measured at the rebars embedded at a cover depth of 20 and 30 mm flatten out after 2 to 3 days, reaching a negligible steady-state cross-sectional loss of less than $1$ µm/year. At either the intermediate or the highest cover depth, there is no significant difference between the measured ${i}_{\text{corr}}$ in samples exposed to tap water and those in contact with concentrated chloride solution. The linear polarization resistance measurements are thus in line with the results of the rapid chloride ingress test (see discussion in Sect. 4.2). The apparent similarity between the corrosion current densities measured in the presence and absence of chlorides raises the question as to whether the exposure time was sufficiently long to allow for chloride-induced corrosion to initiate. With regard to future investigations into the corrosion performance of the MgO/HY binder, it is recommended to repeat the here presented ingress tests using a test solution with a lower pH, i.e. at lower Cl^−^/OH^−^ ratio, to facilitate corrosion initiation [11].

The pore solution pH of MgO-based binders is typically buffered at pH values ranging from 10.5 to 11.0 [3, 17]. In comparison, the pore solution of uncarbonated Portland cement is buffered well above a pH of 12.5 [28]. This high degree of alkalinity, and correspondingly, the thermodynamically favorable stabilization of iron (hydr)oxide phases on the reinforcement surface, is the primary reason as to why the corrosion rate of steel in concrete is negligible [29]. The apparent discrepancy in the degree of alkalinity between these novel and traditional cementitious binders has recently given rise to the hypothesis that MgO-based binders may be unsuited for reinforcement applications [17]. Despite these theoretical considerations, the measured corrosion rates of carbon steel embedded in MgO/HY mortars presented in this study are comparable to or insignificantly higher than the corrosion rate of passive steel embedded in Portland cement [30] as well as steel embedded in carbonated Portland cement [21]. Figure 11 compares various ${i}_{corr}$ here obtained for the 90/10 and 70/30 MgO/HY mix exposed to the ingress of water (w/c = 0.50) to the average corrosion rate of carbon steel embedded in carbonated Portland cement [21] as well as in carbonated CEMI [31, 32] of equivalent w/c, both under fully and partially saturated conditions.

**Fig. 12:**
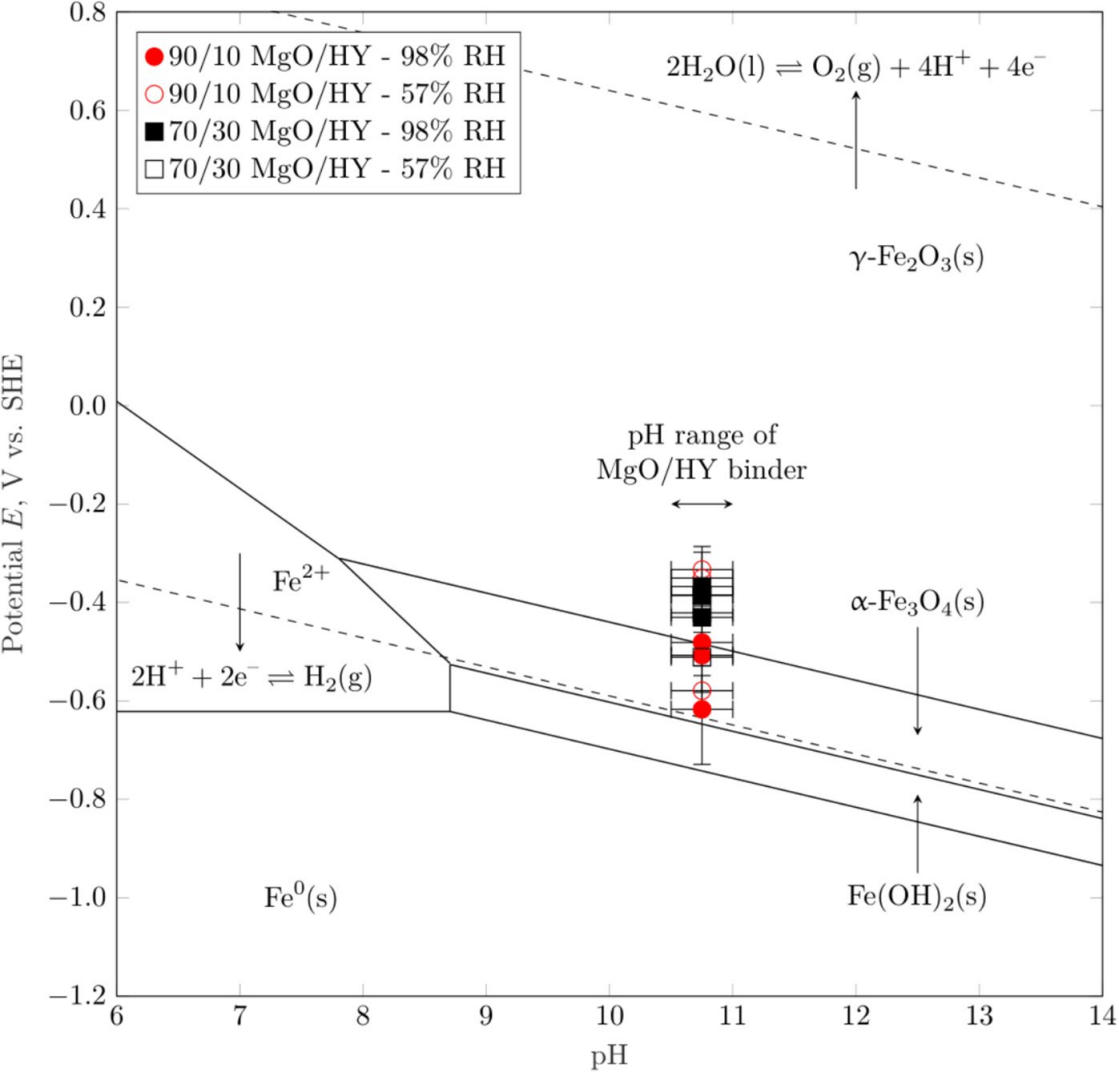
Pourbaix diagram of iron, including selected solid iron (hydr)oxide phases, together with the open circuit potential (OCP) measurements obtained during the corrosion rate measurements presented in this study. The diagram was created using the open source Python library PourPy [33] and the thermodynamic data published in Furcas et al. [34]. The error bar in x (pH) and y (potential) direction represent the pH range of the MgO/HY binder with various MgO-to-HY ratios (by mass) and the standard deviation of 3 OCP measurements for each binder composition and curing condition studied

Figure 12 further displays the Pourbaix diagram of iron, together with the open circuit potential (OCP) measurements obtained during the corrosion rate measurements of carbon steel embedded in the MgO/HY binder, exposed to the ingress of water. The Pourbaix diagram was constructed, using the algorithm outlined in Korber et al. [34]. In short, the potential-independent vertical lines of a generic chemical reaction,

4 $$\sum_{i}{\nu }_{a,i}{A}_{i}+{\nu }_{{\text{H}}^{+}}{\text{H}}^{+}=0,$$

were added to the diagram by computing the pH at which the formation of products is favoured according to.

5 $${\text{pH}}=-{\text{log}}_{10}\left\{{\text{H}}^{+}\right\}=\frac{1}{{\nu }_{{\text{H}}^{+}}}\left[{\text{log}}_{10}\prod_{i}{\left\{{A}_{i}\right\}}^{{\nu }_{a,i}}-{\text{log}}_{10}K\right],$$

where ${\nu }_{{\text{H}}^{+}}$ and ${\nu }_{a,i}$ are the stoichiometric coefficients of the proton as well as all other reactants and products ${A}_{i}$ and $K$ is the equilibrium constant of the reaction. Various lines of all generic potential-dependent electrochemical reactions.

6 $$\sum_{i}{\nu }_{a,i}{A}_{i}+{\nu }_{{\text{H}}^{+}}{\text{H}}^{+}+{\text{ne}}^{-}=0,$$

are plotted via their corresponding Nernst equation.

7 $$E_{rev} = E_{rev}^{^\circ } + 2.303 \times \frac{RT}{{nF}} \times \log_{10} \prod\limits_{i} {\left\{ {A_{i} } \right\}^{{\nu_{a,i} }} } + 2.303 \times \nu_{{H^{ + } }} \times \frac{RT}{{nF}} \times pH,$$

where $n$ is the number of electrons released or consumed, $F=96485.3321$ A mol^−1^ s^−1^ is the Faraday constant, $R=8.31446262$ J mol^−1^ K^−1^ refers to the ideal gas constant and $T$ is the temperature in degree Kelvin.

Across the pH range characteristic to the MgO/HY binder, various OCP measurements are well within the potential-pH stability regime of the solid iron phases white rust (Fe(OH)_2_(s)), magnetite (α-Fe_3_O_4_(s)) and maghemite (γ-Fe_2_O_3_(s)). From a thermodynamic point of view, solely considering the comparatively low pore solution pH, the corrosion of carbon steel in the investigated MgO/HY binder can thus be considered analogous to the corrosion of carbon steel in partially carbonated Portland cement-based concrete. Firstly, both systems feature a pH significantly lower than the pH of uncarbonated Portland cement-based concrete. The pore solution pH of the Mg-based binder investigated in this study is somewhere in between that of fully carbonated and uncarbonated Portland cement-based concrete. Secondly, the measured potentials of carbon steel embedded in the MgO/HY binder, as well as in moist, wet or dry carbonated concrete [35], are in the passivity regime of the classical Pourbaix diagram of iron [18, 29]. Although this overlap suggests that carbon steel embedded in either cementitious system experiences negligible corrosion, it is well known that the instantaneous corrosion rate of steel in carbonated concrete can in fact be technically relevant [31]. As previously detailed in Angst et al. [24], the major influencing factor controlling the degree of corrosion-related damage in carbonated concrete is the moisture state at the steel reinforcement, and therefore, implicitly the concrete cover depth, its pore structure and its integrity (i.e., limited cracking). Similarly, the primary predictor of the corrosion rate of carbon steel embedded in the MgO/HY binder investigated in this study is found to be the mortar cover depth (compare Figs. 9, 10). The satisfactory resistance against the ingress of moisture (compare Figs. 7, 8) suggests that the mortar features a low porosity, sufficient to control the moisture transport through the mortar cover during exposure to liquid water over a duration of 2–3 days. Due to the overall negligible corrosion rates (in the order of $1\times {10}^{-7}$ to $1\times {10}^{-8}$ ${\text{A/cm}}^{2}$ at a low cover depth of 10 mm and $1\times {10}^{-8}$ to $1\times {10}^{-9}$ ${\text{A/cm}}^{2}$ at intermediary cover depths of 20–30 mm), we conclude that the corrosion rate of carbon steel in the MgO/HY binder also primarily depends on the moisture state of the steel reinforcement and that the comparatively low, but still alkaline pore solution pH may be tolerable, as long as the steel–concrete-interface is sufficiently dry [19, 21, 24]. As highlighted previously, further experimental work is needed to assess the long-term corrosion performance of the binder, the corrosion rates of steel exposed to the ingress of aqueous electrolytes featuring a lower Cl^−^/OH^−^ ratio as well as secondary, corrosion-related degradation processes, including cracking and spalling of the concrete cover.

## Conclusion

Results of the rapid chloride ingress test showed high penetration resistance against chlorides of 90/10 and 70/30 MgO/HY mortars prepared with w/c of 0.50 (3.5% SP). Penetration depths were marginal for both mortar mixes ranging between 1 and 5 mm at a total sample length of 50 mm. The associated chloride migration coefficients *D*_*Cl*_ = 0.6 ± 0.3 and 1.0 ± 0.6 · 10^–12^ m^2^/s for the 90/10 and 70/30 mortars, respectively. Although these first screening results are promising, further testing is needed to establish whether the accelerated testing results hold true for natural exposure conditions including the ingress of chlorides due to bulk diffusion and advection.

Formation of Mg-chlorides was not observed by XRD and TGA for 90/10 and 70/30 MgO/HY paste slices cured in alkaline chloride solution for 7 and 28 d. If Mg-chlorides can generally form at the chosen experimental conditions, then the concentration of the chlorides in solution, curing time or penetration depth of the curing solution could have been insufficient. In addition, the high pH of the curing solutions might have impeded the formation of Mg-chlorides.

Corrosion rate measurements further show that carbon steel rods embedded in 90/10 and 70/30 MgO/HY mortars exposed to the capillary ingress of water and chloride-containing solution experience negligible corrosion rates in the orders of $1\times {10}^{-9}$ to $1\times {10}^{-7}$ $\text{A/}{\text{cm}}^{2}$. Across all experiments conducted, the corrosion rate of carbon steel rods with the lowest cover depth of 10 mm, i.e. the ones close to the water table, is one order of magnitude higher than that of the rods embedded at 20 and 30 mm. Findings of these accelerated, short-term experiments suggest that, analogous to the corrosion of steel in carbonated concrete, the moisture content is the main predictor of the corrosion rate of steel in the MgO/HY binder, at least as long as the matrix surrounding the steel remains chloride-free.

The tested MgO/HY binder could prospectively be suitable for use in structural concrete with steel reinforcement. Further investigations are required to assess the long-term corrosion performance of this binder, the role of its microstructure and porosity with regard to the corrosion performance as well as its resistance to other exposure conditions.

## Supplementary Information

Below is the link to the electronic supplementary material.

Supplementary file1 (DOCX 2986 KB)

## Data Availability

The data that support the findings of this study are available from the corresponding author upon reasonable request.
