# Supplementary material for: Durability assessment of MgO/hydromagnesite mortars—Resistance to chlorides and corrosion
Source: Mater Struct. 2025 Sep 20;58(8):266. doi: 10.1617/s11527-025-02765-z (PMC12449408; doi:10.1617/s11527-025-02765-z)
Supplement: Supplementary file 1 — Supplementary file1 (DOCX 2986 KB) [file 11527_2025_2765_MOESM1_ESM.docx]

**Electronic supplementary materials (ESM)**

# Characterization of raw materials


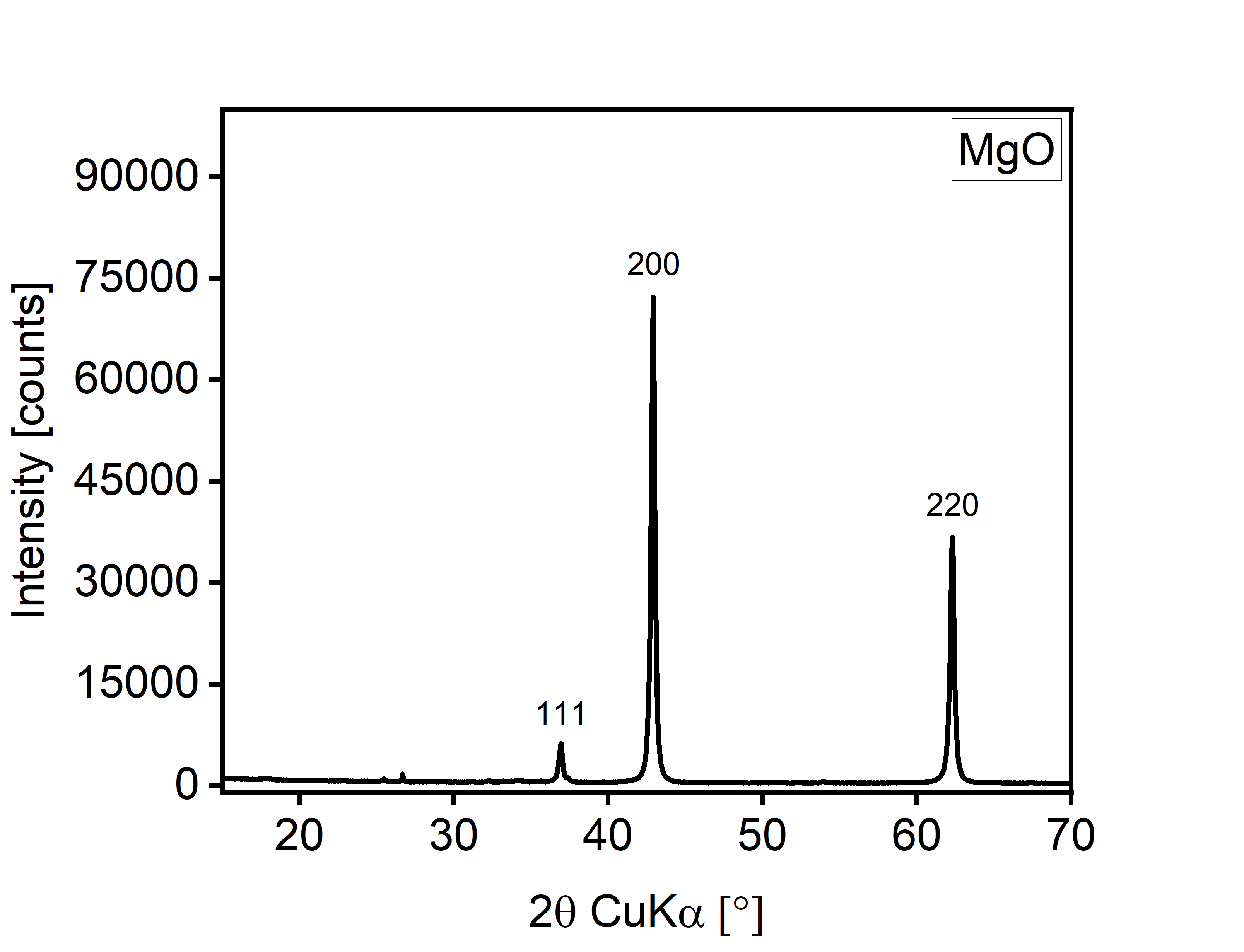


**Fig. A1**: Diffraction pattern of reactive MgO powder. Reflections are indexed according to PDF 01-071-1176 [1].


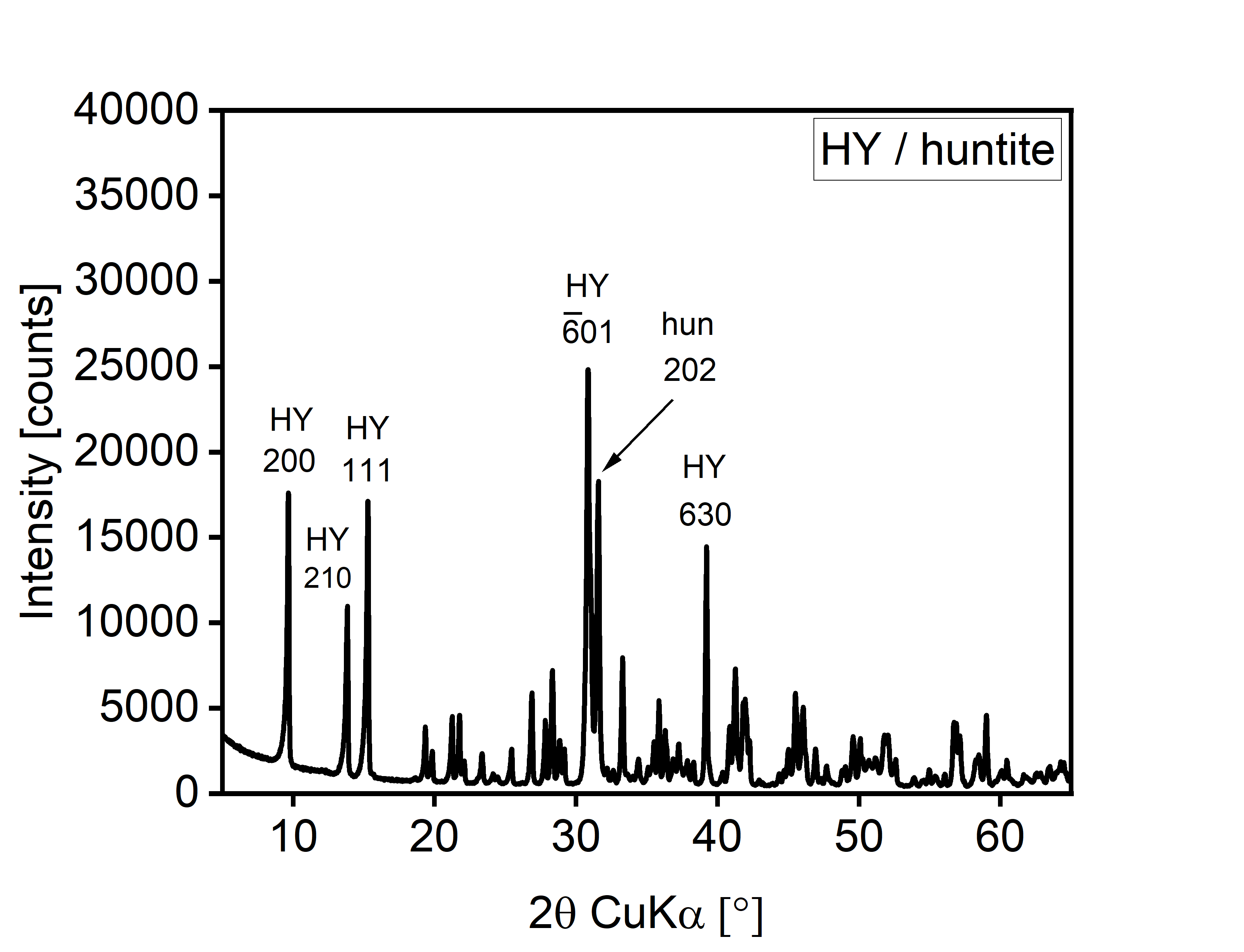


**Fig. A2**: Diffraction pattern of the natural HY/huntite mix. Major reflections of HY and huntite are indexed according to PDF 00-008-0179 [2].


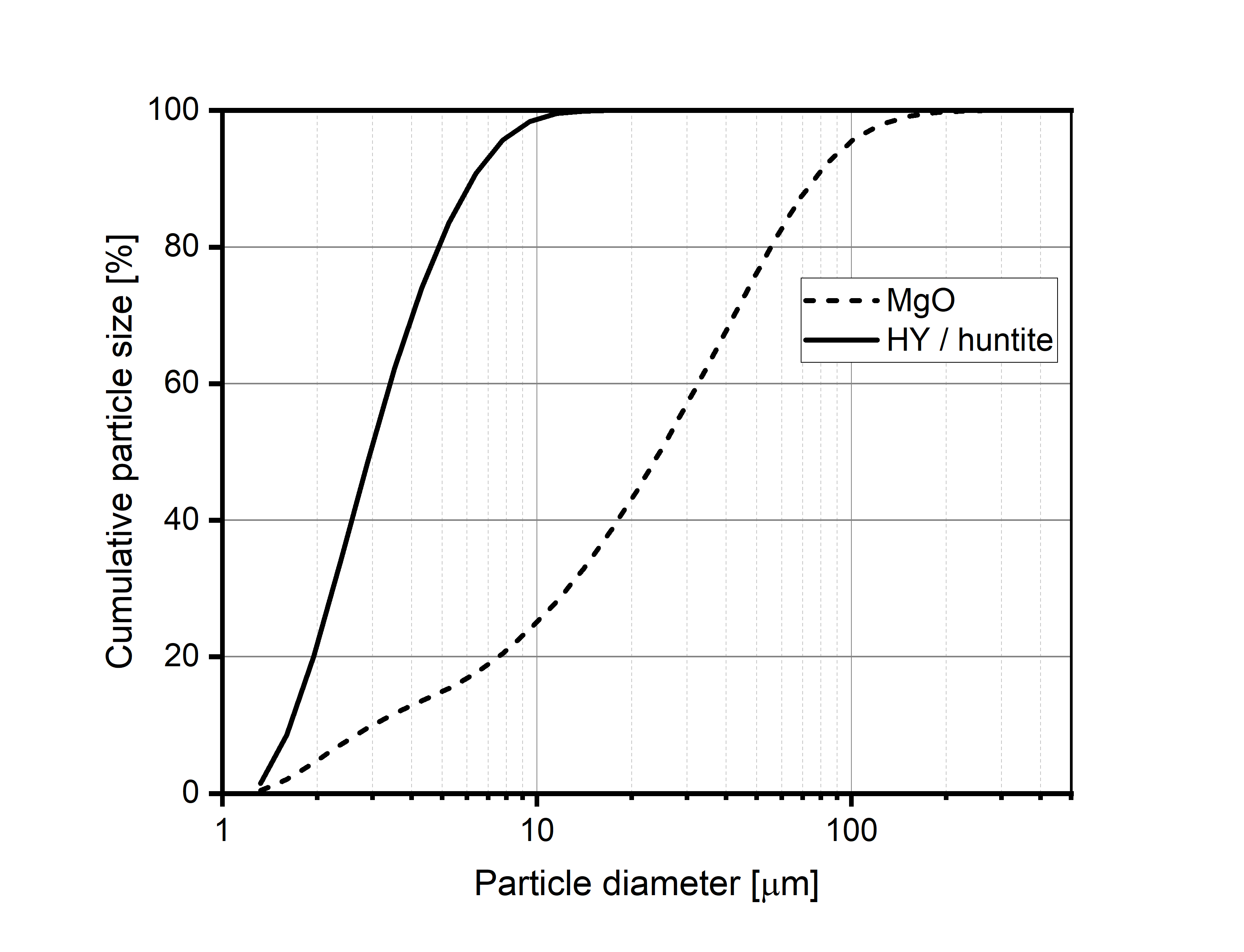


**Fig. A3**: Cumulative particle size distribution of reactive MgO and the natural HY/huntite mix.

# Rapid chloride ingress test setup


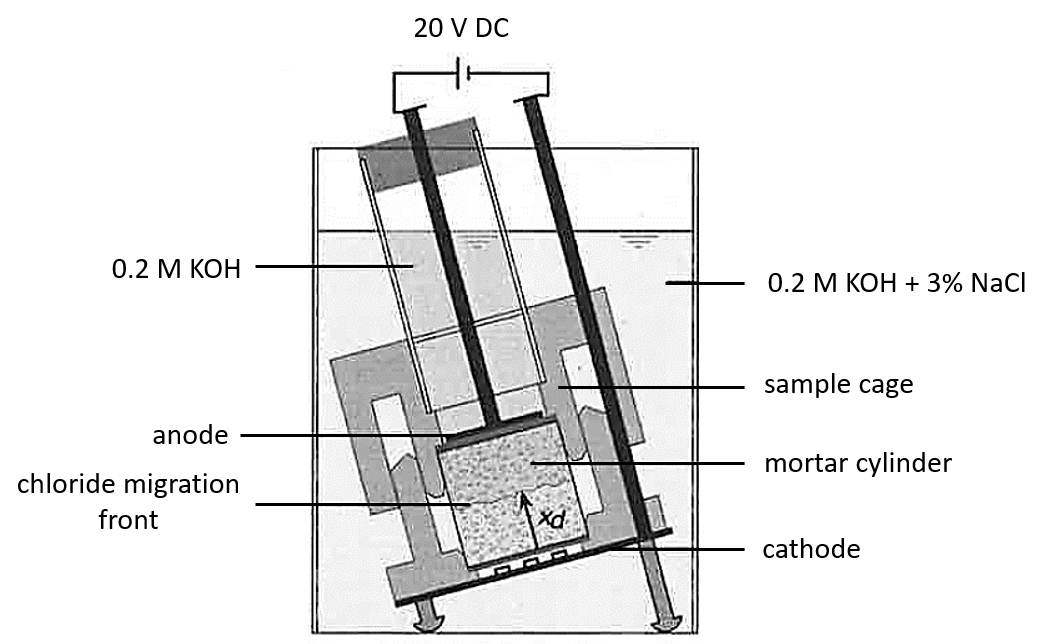


**Fig. A4**: Chloride migration cell sketch. Modified after SIA 262/1 Appendix B.

# Phase analysis of pastes


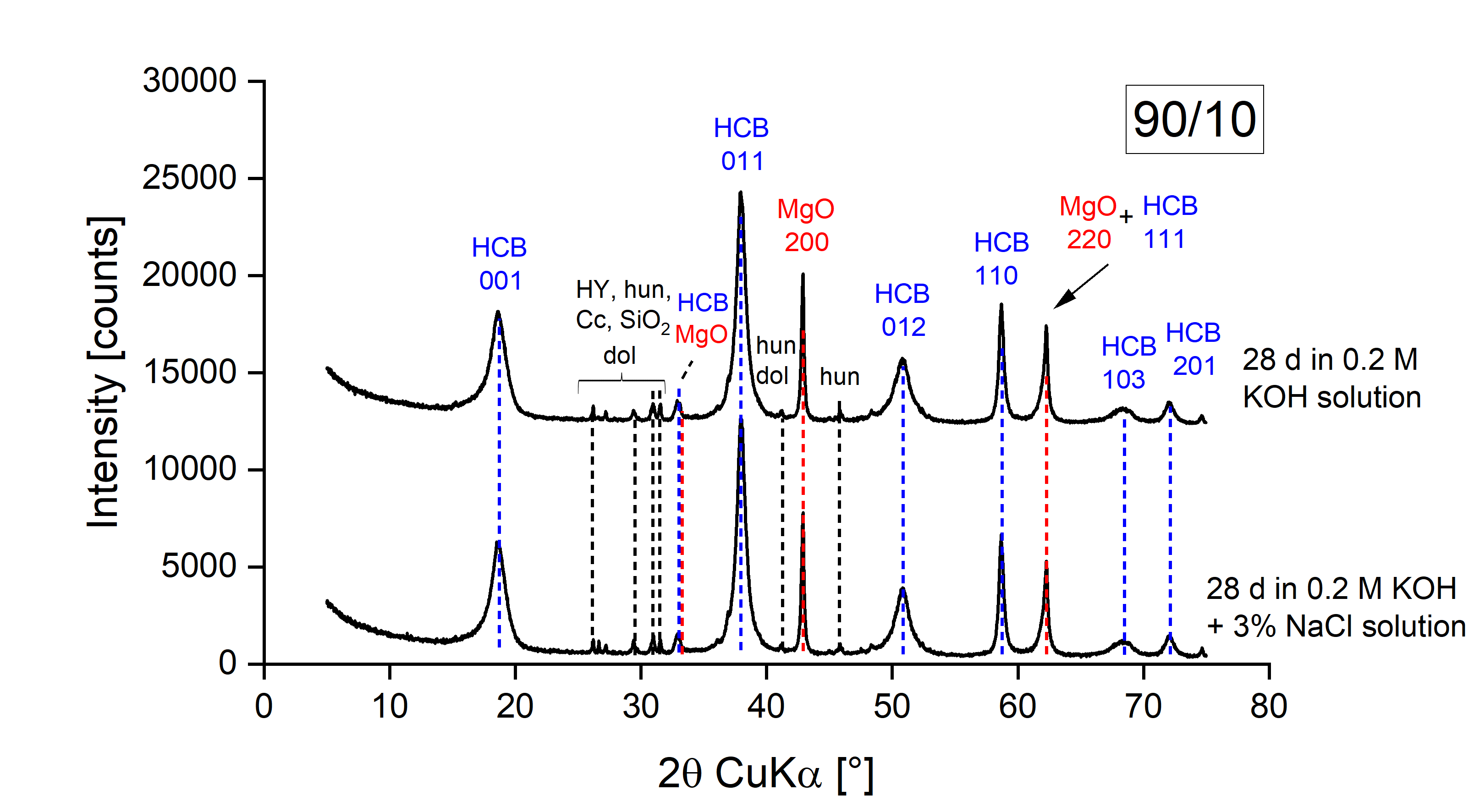


**Fig. A5**: Diffraction patterns of a 90/10 paste cured at 98% RH for 28 d and afterwards additionally cured in an alkaline solution (0.2 M KOH) with and without 3% NaCl for 28 d.


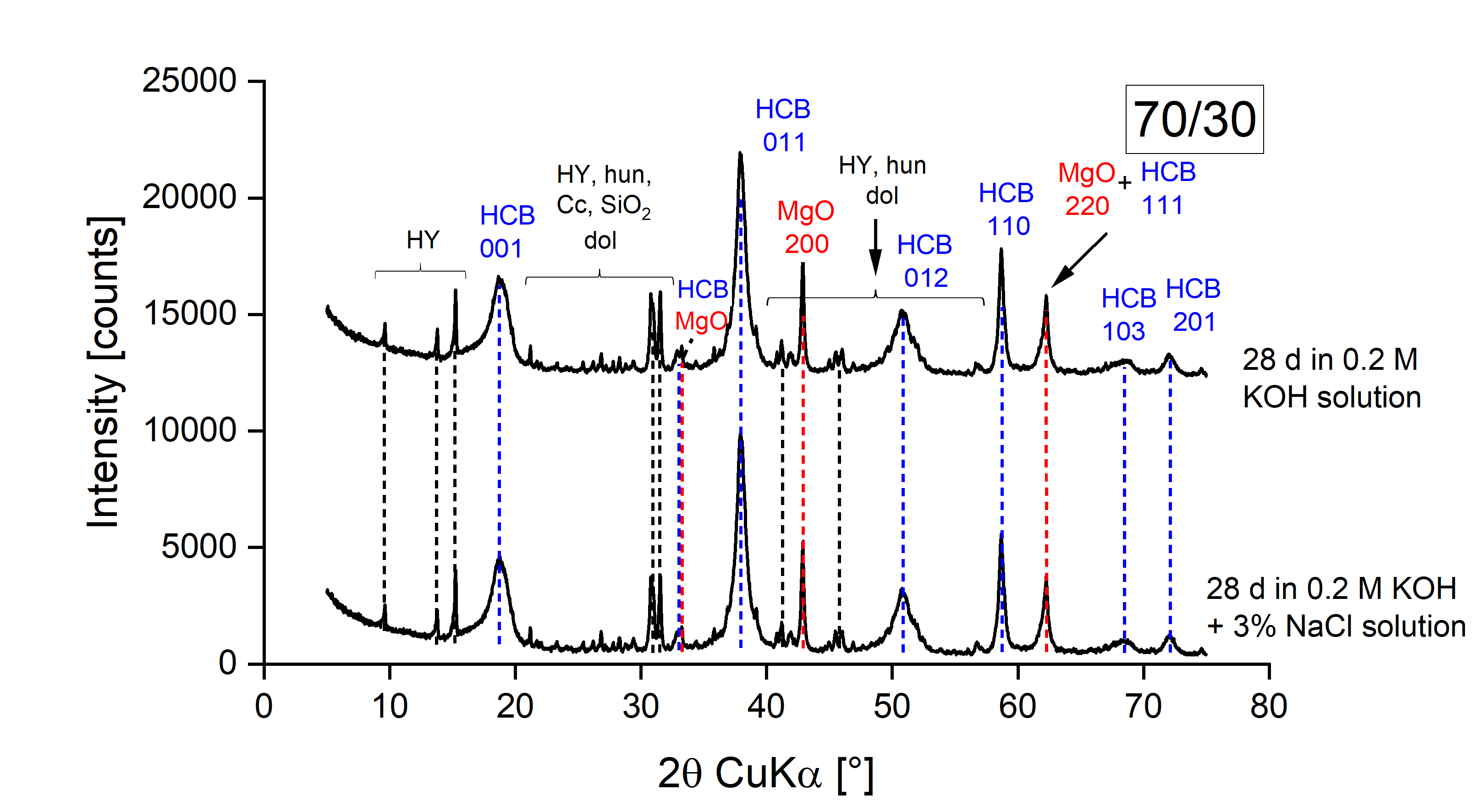


**Fig.** **A6**: Diffraction patterns of a 70/30 paste cured at 98% RH for 28 d and afterwards additionally cured in an alkaline solution (0.2 M KOH) with and without 3% NaCl for 28 d.


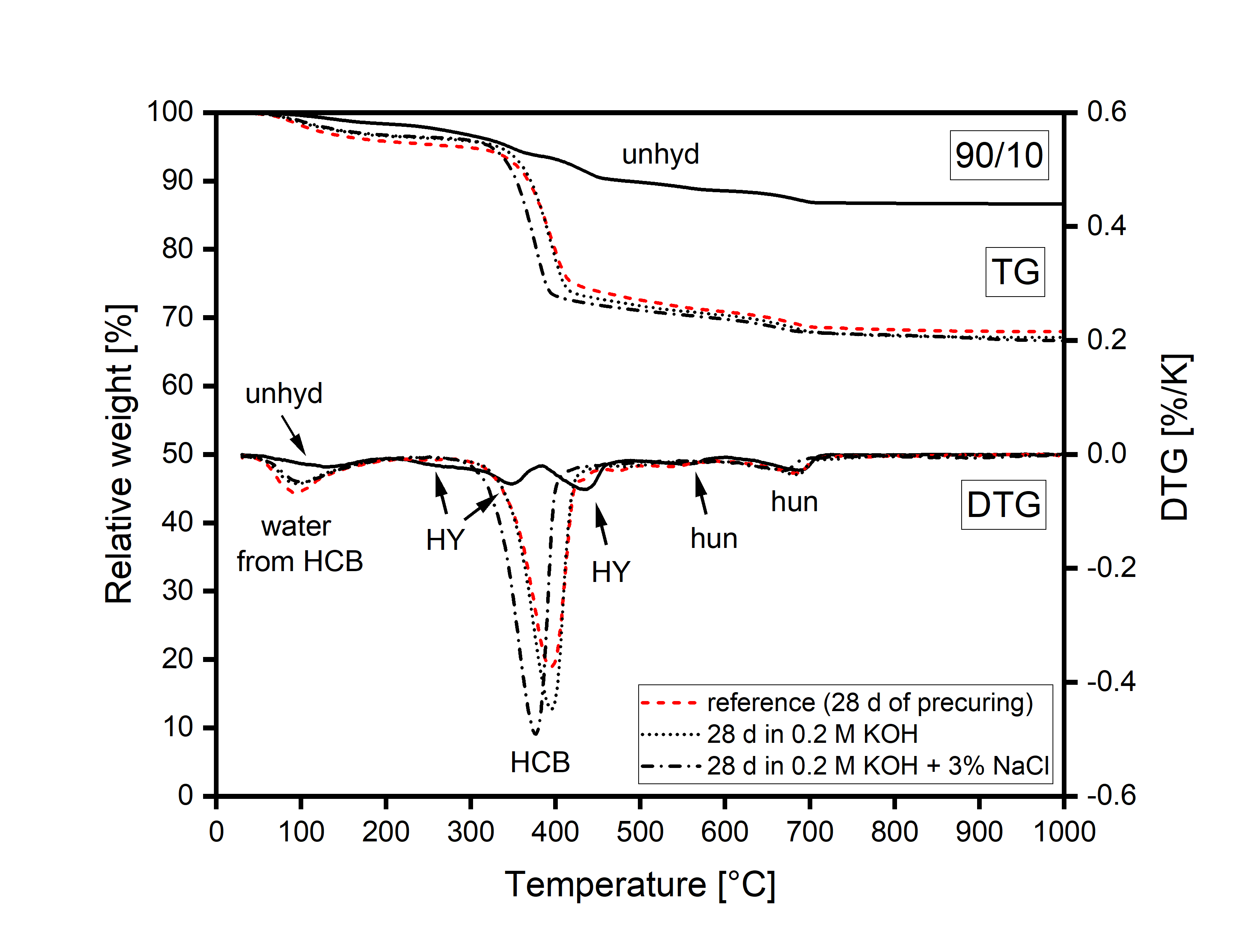


**Fig. A7**: TGA data of an unhydrated 90/10 binder and a 90/10 paste cured at 98% RH for 28 d (reference, marked red) and afterwards additionally cured in an alkaline solution (0.2 M KOH) with and without 3% NaCl for 28 d.


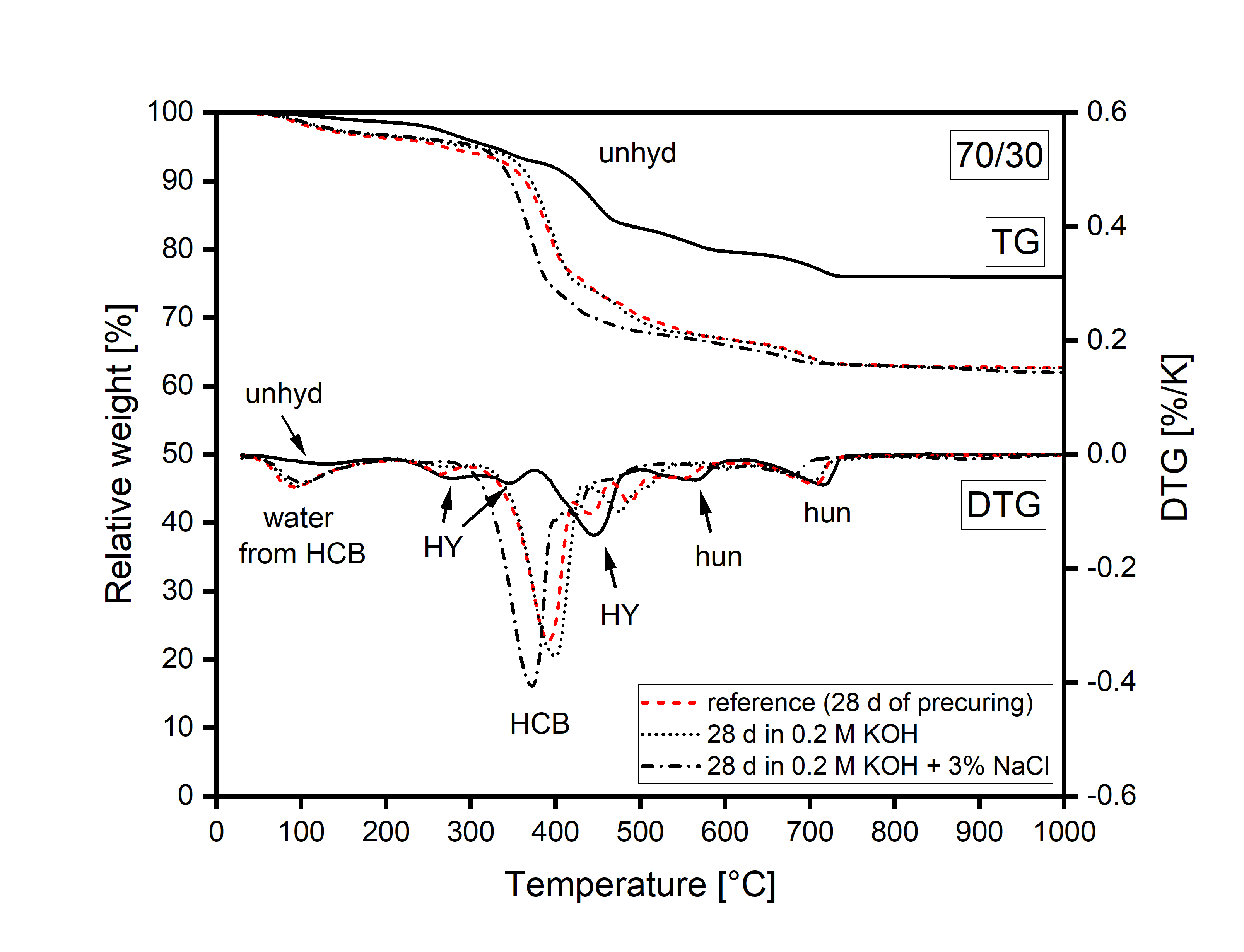


**Fig.** **A8**: TGA data of an unhydrated 70/30 binder and a 70/30 paste cured at 98% RH for 28 d (reference, marked red) and afterwards additionally cured in an alkaline solution (0.2 M KOH) with and without 3% NaCl for 28 d.

# Rapid chloride migration test


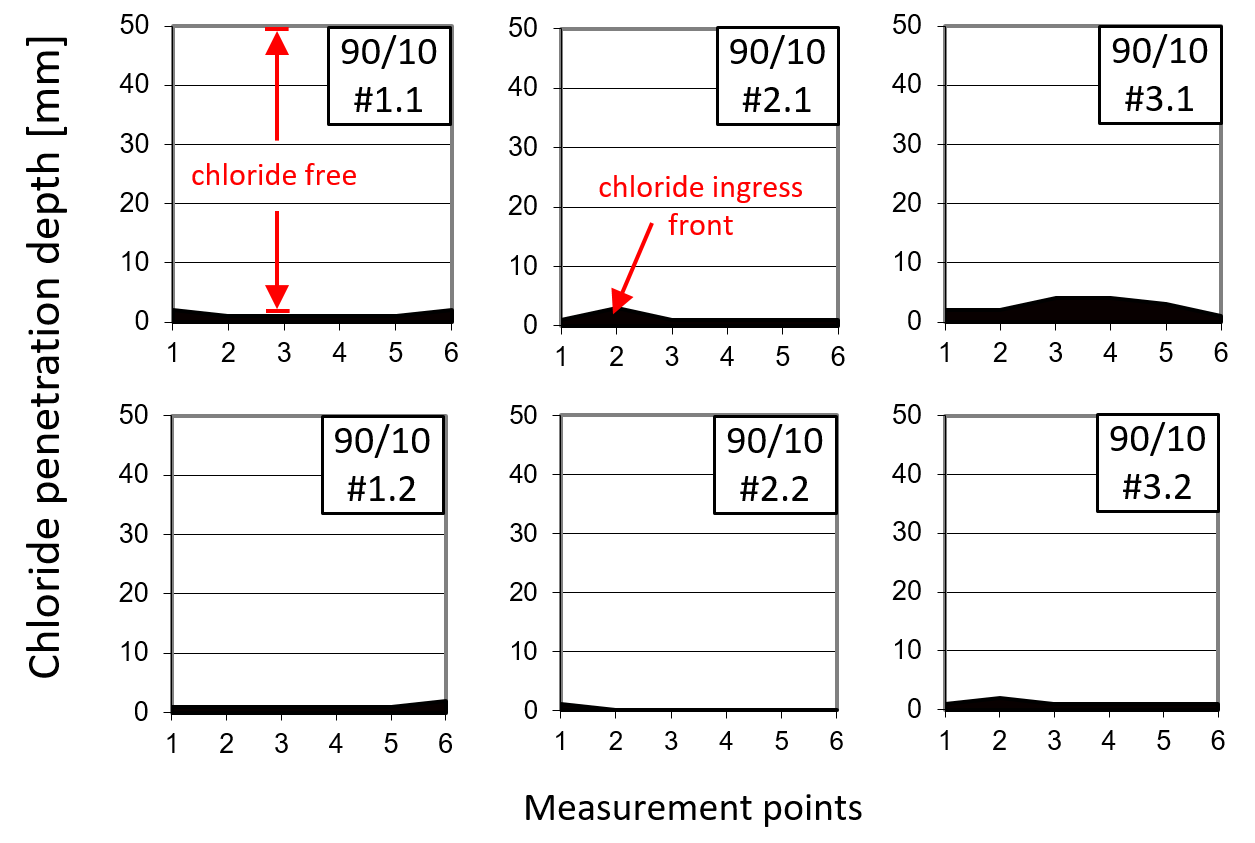


**Fig. A9**: Chloride penetration depth measured on three split, cylindrical 90/10 mortar samples. Example for diagram labels: (#1.1): split first halve of the first sample, (#1.2): split second halve of the first sample, etc.


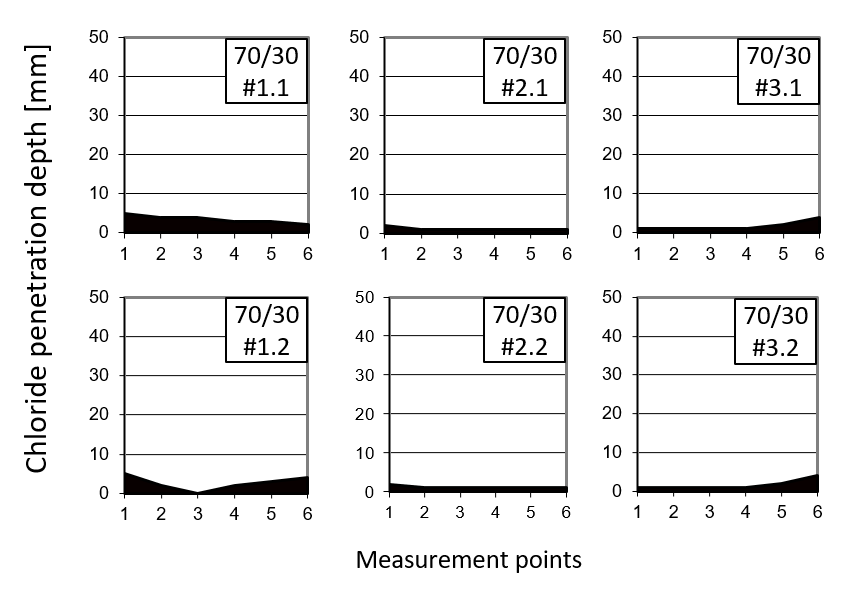


**Fig. A10**: Chloride penetration depth measured on three split, cylindrical 70/30 mortar samples.


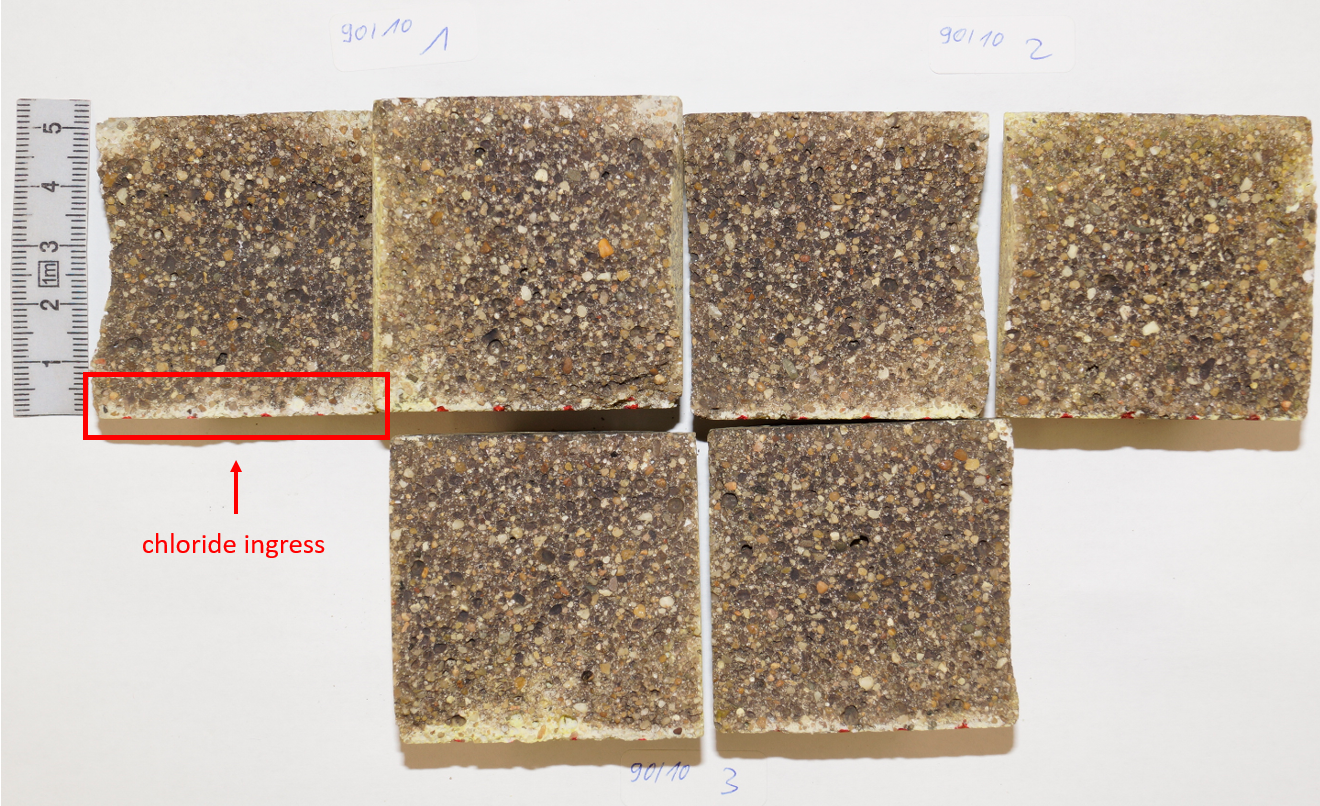


**Fig. A11**: Pictures of split 90/10 MgO/HY mortar halves used for chloride migration tests after SIA 262/1 Appendix B. Samples were dyed with 0.1% fluorescein solution (ethanol as solvent) and 0.1 N silver nitrate solution. Bright areas mark chloride ingress areas.

## References

[1] S. Sasaki, K. Fujino, Y. Takeuchi, X-Ray Determination of Electron-Density Distributions in Oxides, MgO, MnO, CoO, and NiO, and Atomic Scattering Factors of their Constituent Atoms, Proc. Jpn. Acad., 55 (1979) 43.

[2] J. Murdoch, Unit Cell of Hydromagnesite, Am. Miner., 39 (1954) 24-29.
